# Antisense oligonucleotides modulate aberrant inclusion of poison exons in *SCN1A*-related Dravet syndrome

**DOI:** 10.1172/jci.insight.188014

**Published:** 2025-02-13

**Authors:** Sheng Tang, Hannah Stamberger, Jeffrey D. Calhoun, Sarah Weckhuysen, Gemma L. Carvill

**Affiliations:** 1 Department of Neurology, Feinberg School of Medicine, Northwestern University, Chicago, Illinois, USA.; 2 Applied & Translational Neurogenomics Group, VIB Center for Molecular Neurology, VIB, Antwerp, Belgium.; 3 Translational Neurosciences, Faculty of Medicine and Health Science, University of Antwerp, Antwerp, Belgium.; 4 Department of Neurology, Antwerp University Hospital, Antwerp, Belgium.

**Keywords:** Genetics, Neuroscience, Epilepsy, Gene therapy

## Abstract

Dravet syndrome is a developmental and epileptic encephalopathy associated with pathogenic variants in *SCN1A*. Most disease-causing variants are located within coding regions, but recent work has shed light on the role of noncoding variants associated with a poison exon in intron 20 of *SCN1A*. Discovery of the *SCN1A* poison exon known as 20N has led to the first potential disease-modifying therapy for Dravet syndrome in the form of an antisense oligonucleotide. Here, we demonstrate the existence of 2 additional poison exons in introns 1 and 22 of *SCN1A* through targeted, deep-coverage long-read sequencing of *SCN1A* transcripts. We show that inclusion of these poison exons is developmentally regulated in the human brain, and that deep intronic variants associated with these poison exons lead to their aberrant inclusion in vitro in a minigene assay or in iPSC-derived neurons. Additionally, we show that splice-modulating antisense oligonucleotides can ameliorate aberrant inclusion of poison exons. Our findings highlight the role of deep intronic pathogenic variants in disease and provide additional therapeutic targets for precision medicine in Dravet syndrome and other *SCN1A*-related disorders.

## Abstract 2

Dravet syndrome-related variants increase aberrant splicing of poison exons in SCN1A, and rationally designed ASOs can ameliorate pathologic splicing in patient iPSC-derived neurons.

## Graphical abstract

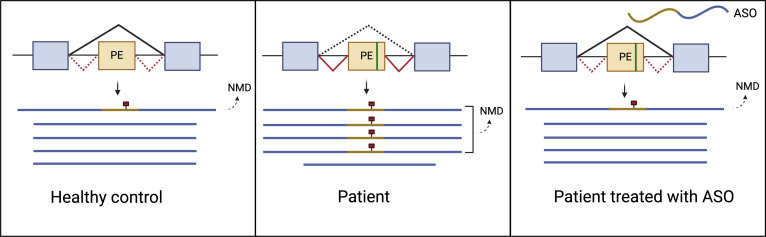

## Introduction

The developmental and epileptic encephalopathies (DEEs) are a group of rare epilepsy syndromes characterized by early-onset seizures and developmental delay (1). Among the DEEs, Dravet syndrome is considered a prototypic genetic epilepsy given its prevalence and genotype-phenotype correlation (2, 3). At least 80% of Dravet syndrome is associated with pathogenic variants in *SCN1A*, with a minority of cases unsolved.

Recent work has highlighted the role of noncoding variation in Dravet syndrome by describing the existence of a poison exon in intron 20, termed 20N (4). Poison exons are alternatively spliced exons that when included in a transcript, introduce a premature termination codon (PTC), which usually targets the transcript for nonsense-mediated decay (NMD) (5). Genetic epilepsy with febrile seizures plus (GEFS+) or Dravet syndrome–related variants in the 20N region were shown to increase its inclusion, providing a mechanism for *SCN1A* haploinsufficiency for these patients (6). Subsequently, 20N was developed as a therapeutic target for antisense oligonucleotides (ASOs), which increased the abundance of the corresponding protein, voltage-gated sodium channel subunit Na_V_1.1, by reducing the unproductive splicing of 20N (7, 8). This therapy is currently in clinical trials as the first potential disease-modifying treatment for Dravet syndrome (ClinicalTrials.gov NCT04740476).

Despite the advances in our understanding of the genetics of Dravet syndrome and other DEEs, many cases remain unsolved. Specifically, deep intronic variants are usually considered variants of uncertain significance, confounding genetic diagnosis and hindering disease-specific treatment. Recent studies have shown that poison exons are under-annotated and are a potentially important source of disease-causing variants in *SCN1A* and other genes related to neurodevelopmental disorders (9).

Here, we studied 2 noncoding variants in *SCN1A*, both of which were previously discovered in patients with Dravet syndrome (9). Using targeted, deep-coverage, long-read sequencing of *SCN1A* transcripts, we demonstrate the existence of 2 poison exons in introns 1 and 22 of *SCN1A*. We show that poison exons are developmentally regulated in the human brain, and that Dravet syndrome–related variants located in these poison exons cause their aberrant inclusion. Additionally, we show that *SCN1A* poison exon inclusion is sensitive to NMD, supporting their role for negative regulation of *SCN1A* transcript abundance. Finally, we demonstrate the capability of splice-modulating ASOs to ameliorate aberrant *SCN1A* poison exon inclusion in a disease context and achieve a substantial degree of allele specificity.

## Results

### SCN1A noncoding variants are associated with poison exons in introns 1 and 22.

Previous work described 2 noncoding, deep intronic variants found in patients with Dravet syndrome (9). These variants, located in introns 1 and 22, were predicted to be pathogenic through their association with putative poison exons in these regions (Table 1). Given that Dravet syndrome is typically associated with *SCN1A* haploinsufficiency, we hypothesized that these variants would increase the aberrant inclusion of poison exons in these regions.

In the literature, the term “poison exon” is sometimes used interchangeably with the terms “cryptic exon” and “pseudoexon.” In this study, we use a working definition of a poison exon with the following criteria: (a) it is an alternatively spliced exon flanked by distinct donor and/or acceptor splice sites, which distinguishes it from retained introns; (b) its inclusion into the transcript introduces a PTC, which distinguishes it from other alternatively spliced cassette exons; and (c) it is naturally occurring. Poison exons can also be associated with pathogenic variants that lead to their aberrant inclusion in disease contexts (4, 10, 11).

**Figure 1:**
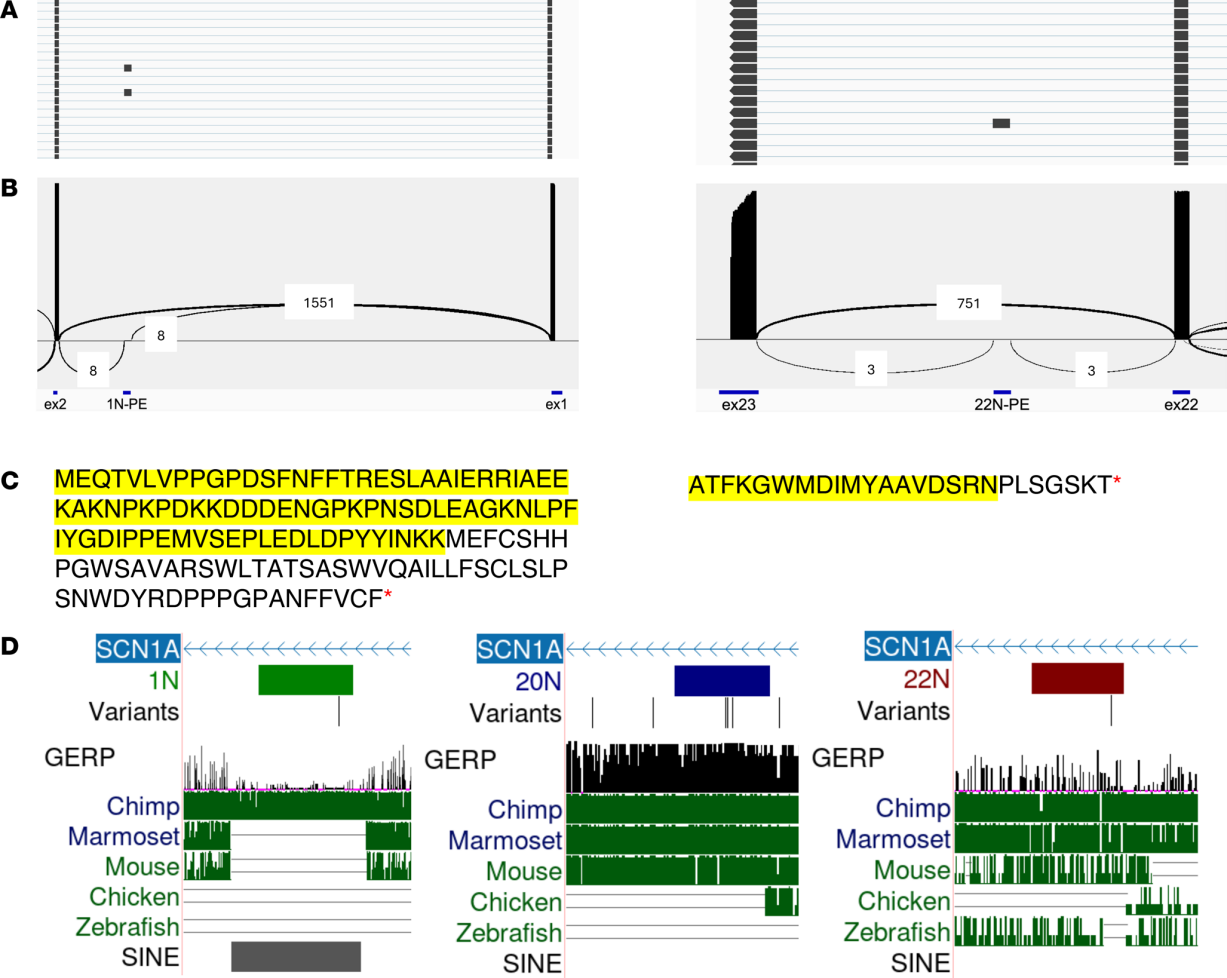
Features and conservation of *SCN1A* poison exons. (**A**) Targeted RT-PCR and long-read sequencing of *SCN1A* transcripts in control iNeurons shows rare inclusion of alternatively spliced exons in intron 1 (left) and intron 22 (right). Shown are representative views of reads displayed in Integrative Genomics Viewer. Note that *SCN1A* is located on the negative (–) strand of chromosome 2, and therefore its sequence is displayed from right to left. (**B**) Sashimi plots showing alternative splicing of 1N (left) and 22N (right) in iNeurons from healthy controls. PE, poison exon. (**C**) In-frame in silico translation starting from the nearest 5′ canonical exon shows that 1N (left) and 22N (right) each introduce a PTC (red asterisk) when spliced into the reading frame, suggesting they could serve as poison exons. The portion of the translation identical to the canonical SCN1A protein sequence is highlighted, with the unhighlighted portion representing the contribution of the poison exon. (**D**) Poison exons, associated pathogenic variants, and conservation. The poison exons, their related variants, the GERP score, and UCSC conservation track (Multiz alignments for vertebrate species) are shown. The GERP score is an estimate of evolutionary constraint (scale: 0 to 7; positive scores suggest evolutionary constraint) (40). From left to right: 1N is poorly conserved and overlaps with a SINE (gray bar), suggesting that it may have occurred due to a recent retrotransposition event during primate evolution. 20N, previously described, is well conserved in most mammalian species. 22N only shows limited conservation in primates.

By combining targeted reverse transcription PCR (RT-PCR) and deep-coverage long-read sequencing of *SCN1A* transcripts, we first investigated the inclusion of putative poison exons in healthy control induced pluripotent stem cell–derived (iPSC-derived) neurons (iNeurons). In contrast with whole-transcriptome RNA-seq datasets, in which low-abundance *SCN1A* transcripts are difficult to detect, our targeted approach yielded hundreds to thousands of reads mapping to *SCN1A* in each sample, allowing a detailed view of the splicing isoforms of the *SCN1A* transcript. In iNeurons from healthy controls, we found rare but consistently detectable inclusion of 2 exons in introns 1 and 22, respectively (Figure 1, A and B). Importantly, these exons were flanked by the donor and acceptor splice sites GT and AG, respectively, without evidence of intron retention. In silico translation showed that each of these exons, when spliced into the canonical *SCN1A* transcript, introduced a PTC (Figure 1C). These features, the presence of distinct donor and acceptor splice sites and the introduction of a PTC, are shared with the known *SCN1A* poison exon 20N, which we also detected in our long-read sequencing. Together, these findings suggest that these cassette exons serve as poison exons that could potentially target their parent mRNA transcripts for NMD. Hereon, we will refer to them as 1N and 22N (“N” referring to nonsense).

1N and 22N show varying degrees of conservation (Figure 1D). In contrast with the previously described 20N (4), which is well conserved in mammals, 22N is only conserved in some mammalian species, and 1N is not conserved in nonprimate species. Interestingly, 1N lies within a known short-interspersed nuclear element (SINE) whose location is unique in a subset of primates, suggesting that this poison exon may have arisen from a recent retrotransposition event during evolution. In addition, whereas 20N and 22N have fixed lengths, 1N appears to be polymorphic, with multiple alternative 3′ splice donor sites, although all give rise to a PTC (Table 2).

### Developmental regulation of SCN1A poison exons and their sensitivity to NMD.

**Figure 2:**
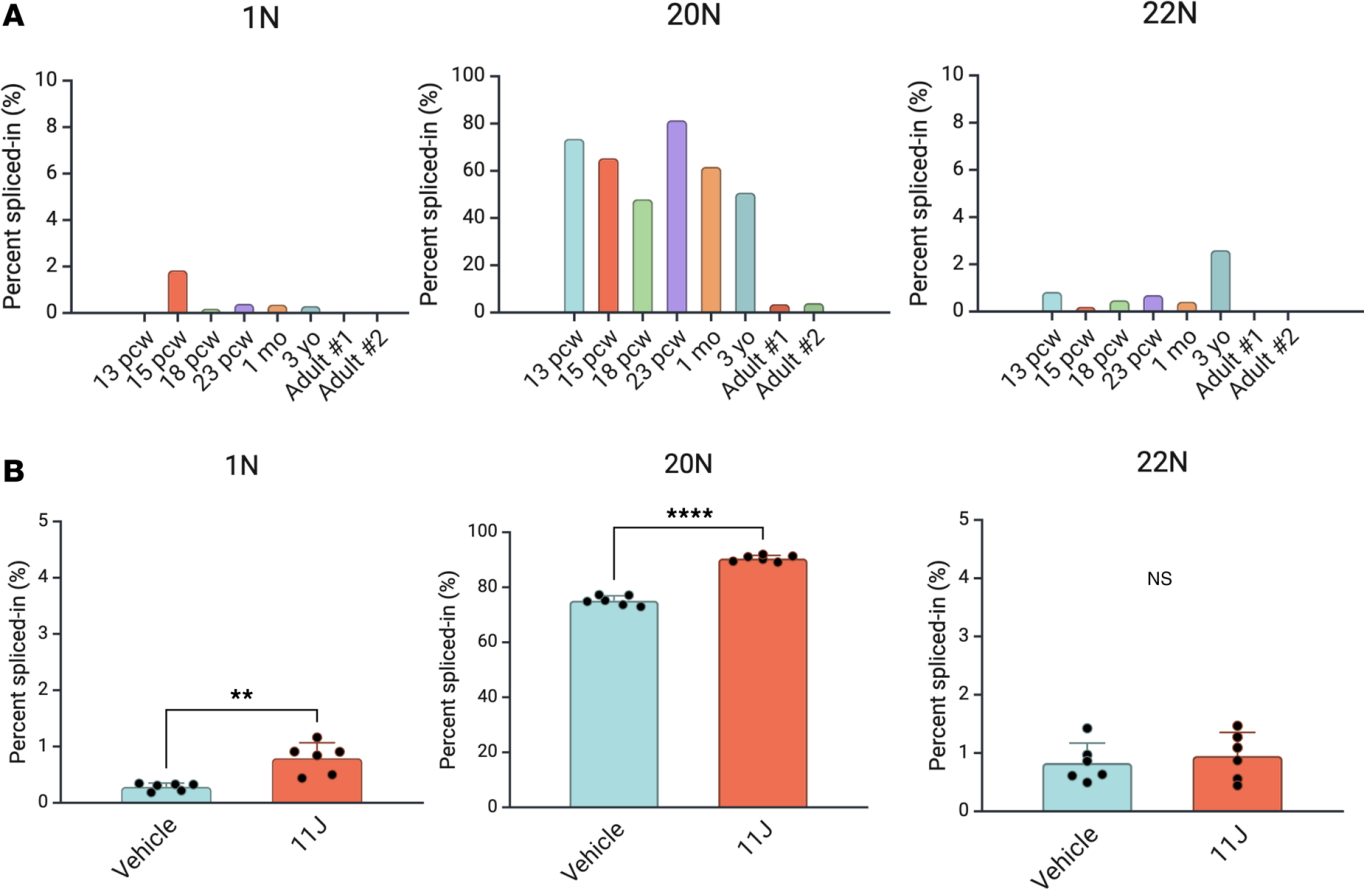
Developmental regulation of *SCN1A* poison exons and NMD sensitivity in iNeurons. (**A**) Developmental regulation of *SCN1A* poison exons. Targeted RT-PCR followed by long-read sequencing was used to assess relative inclusion of 1N, 20N, and 22N across fetal and postnatal development in the human cerebral cortex. 20N inclusion was high throughout the prenatal period and was downregulated after the first several postnatal years. In contrast, 1N and 22N inclusion was relatively low (<5%) prenatally, but still showed a trend of downregulation postnatally. Note that for display purposes, 1N and 22N are shown on a different *y*-axis scale. pcw, postconception weeks. *n* = 1 human brain sample per developmental time point, and at least 500 reads per sample were used to calculate PSI in each sample. (**B**) Relative inclusion of 1N, 20N, and 22N in iNeurons from healthy controls with or without NMD inhibition with 11J. 1N and 20N, but not 22N, show significantly increased relative inclusion after 4 hours of treatment with 1 μM 11J. Note that for display purposes, 1N and 22N are shown on a different *y*-axis scale. *n* = 6 biological replicate wells of iNeurons per condition, and at least 500 reads per sample were used to calculate PSI in each sample. **P < 0.01, ****P < 0.0001 by unpaired *t* test.

Together with the previously described 20N (4), the poison exons 1N and 22N represent a triad of noncoding elements in *SCN1A* that could explain the pathogenic mechanism of deep intronic variants in this gene. Whether these poison exons serve a physiologic role during neural development, however, remains unclear. To investigate whether these poison exons are developmentally regulated, we assessed their relative abundance during human prenatal and postnatal development in samples of cerebral cortex from healthy controls (Figure 2A and Supplemental Figure 1; supplemental material available online with this article; https://doi.org/10.1172/jci.insight.188014DS1). Of the 3 poison exons, we found that 20N is spliced into the *SCN1A* transcript at high levels throughout fetal brain development and in the early postnatal years and is subsequently markedly downregulated in the adult brain. These findings are consistent with the results of previous studies in animal models (12). In contrast, 1N and 22N are spliced in at relatively low levels throughout development but also appear to show a slight downregulation in adulthood. Together, these results show that 20N is the dominant poison exon in *SCN1A* transcripts in the developing brain and that in healthy control brains, 1N and 22N are included in a small fraction of mature *SCN1A* transcripts.

Given that poison-exon-containing transcripts typically undergo NMD, we next tested whether transcripts containing 1N, 20N, and 22N poison exons were sensitive to inhibitors of this process. We applied 11J (an inhibitor of SMG1, a key component of NMD machinery; refs. 13, 14) to iNeurons from healthy controls. Interestingly, both 1N and 20N, but not 22N, showed an upregulation of relative inclusion after 11J treatment (Figure 2B).

### SCN1A 22N–related variant increases poison exon inclusion in a minigene assay.

**Figure 3:**
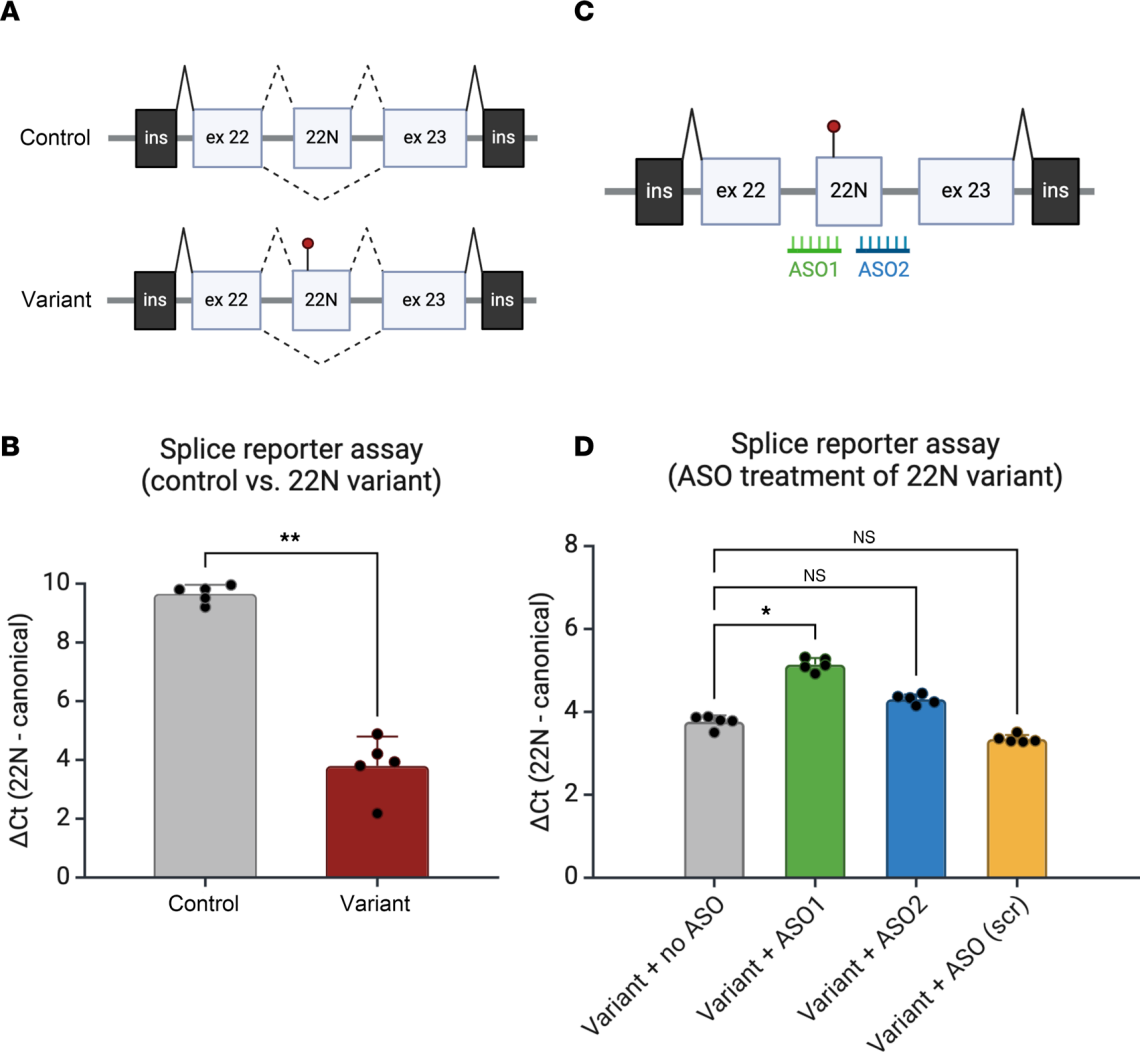
An in vitro minigene assay demonstrates aberrantly increased inclusion of 22N due to a Dravet syndrome–related variant. (**A**) Generation of a splice reporter assay containing intron 22 flanked by canonical exons 22 and 23, containing the putative 22N poison exon. The minigene was inserted into a splice reporter plasmid and transfected into HEK293T cells. Note the presence of rat insulin exons, which serve as constitutively spliced control exons. The position of the Dravet syndrome–related variant is indicated on the variant construct. Created with BioRender. (**B**) Increased inclusion of 22N due to the presence of a Dravet-related variant in 22N (*n* = 5 biological replicate transfections for each of control- or variant-transfected cells). The 22N-related variant leads to decreased ΔCt (22N – canonical splice product), which represents a higher relative abundance of the 22N-containing splice product. (**C**) Design of splice-switching ASOs for 22N. ASO1 targets the acceptor splice site with the 3′ end of the upstream intron, whereas ASO2 targets the donor splice site with the 5′ end of the downstream intron. Created with BioRender. (**D**) Splice-switching ASOs ameliorate aberrantly increased inclusion of 22N. qRT-PCR data with 22N-containing splice product normalized to canonical splice product. In contrast with untreated cells or cells treated with a scrambled ASO, ASO1 and to a lesser extent, ASO2, both reduced aberrant inclusion of 22N. ASO1-treated cells demonstrate a higher ΔCt (22N – canonical splice product) compared with untreated cells, which represents a lower relative abundance of the 22N-containing splice product (*n* = 5 biological replicate transfections for each condition). Significance was assessed by Mann-Whitney test (**B**) or Kruskal-Wallis test with Dunn’s multiple-comparison test (**D**). *P < 0.05, **P < 0.01 (**B**: *P* = 0.0079, no ASO vs. ASO1; **D**: *P* = 0.038, no ASO vs. ASO2). NS, not significant for no ASO vs. ASO2 and no ASO vs. ASO (scr).

To investigate the effect of poison-exon-related variants on the alternating splicing of *SCN1A*, we first developed a splice reporter assay for the 22N poison exon. Using a previously described approach (4, 6), we constructed a minigene containing *SCN1A* canonical exons 22 and 23 (Figure 3A), with the intervening intronic sequence, which contains the putative 22N. We found that compared with control, a minigene carrying the 22N-related patient variant resulted in significantly increased inclusion of 22N relative to the canonical splice product (Figure 3B).

Given the promise of ASO therapy in Dravet syndrome and other neurodevelopmental disorders (8, 15, 16), we next asked whether ASOs targeting 22N could modulate pathologic splicing caused by the 22N patient variant. We designed and tested 2 ASOs targeting the 5′ and 3′ splice junctions (Figure 3C). Of note, all ASOs in this study were designed to have full phosphorothioate linkages with 2′-*O*-methoxyethyl (2′-MOE) modifications throughout, to support steric blockade and facilitate exon skipping or splice switching of the poison exon, while minimizing undesirable RNase H activity (17). Compared with untreated cells or cells treated with a scrambled ASO (ASO1), which targets the 5′ splice junction, significantly reduced the abundance of 22N-containing splice products relative to the canonical isoform, suggesting that it had a favorable effect on modulating splicing (Figure 3D and Supplemental Figure 2). ASO2 showed a similar trend, although this was not statistically significant. Taken together, we show that a Dravet-related variant in 22N increased the inclusion of this poison exon, and that rationally designed, splice-switching ASOs can ameliorate pathologic splice patterns.

### SCN1A 1N–related variant increases poison exon splicing in patient iNeurons and modulation by ASOs.

**Figure 4:**
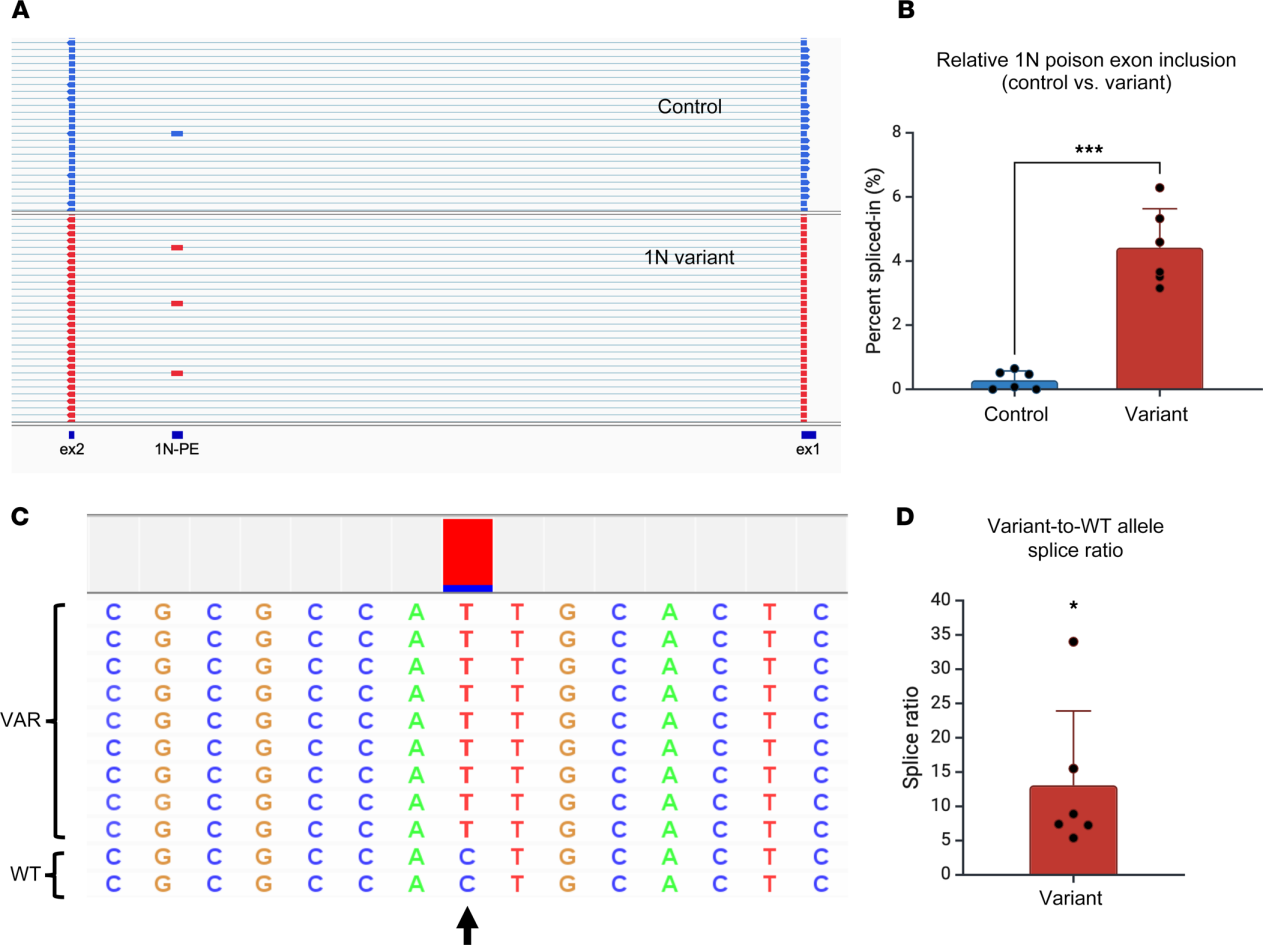
Aberrantly increased inclusion of 1N in iNeurons carrying a Dravet syndrome–related variant. (**A**) Sample reads from targeted RT-PCR and long-read sequencing of *SCN1A* transcripts in iNeurons derived from a healthy control or a patient with Dravet syndrome carrying a variant within 1N. Shown are representative views of reads displayed in Integrative Genomics Viewer. PE, poison exon. (**B**) Targeted *SCN1A* RT-PCR and long-read sequencing shows increased inclusion of 1N in patient compared with control iNeurons (*n* = 5 biological replicate wells of iNeurons per genotype). Each sample had at least 1000 reads from which PSI (%) was calculated. (**C**) A sample set of reads containing 1N obtained from heterozygous patient iNeurons. The position of the variant is indicated by the arrow, with the color map above the position showing a skewed ratio of reads containing T (pathogenic variant, in red) versus C (WT variant, in blue). For this sample, there were a total 37 variant alleles to 5 WT alleles containing 1N, resulting in a splice ratio of 7.4, showing that increased 1N inclusion is strongly skewed toward the allele carrying the pathogenic variant. (**D**) Increased variant-to-WT allele splice ratio in reads containing 1N in patient iNeurons (*n* = 6 biological replicate wells of iNeurons). *P < 0.05; ***P < 0.001 by unpaired *t* test (**B**) or 1-sample *t* test (**D**).

**Figure 5:**
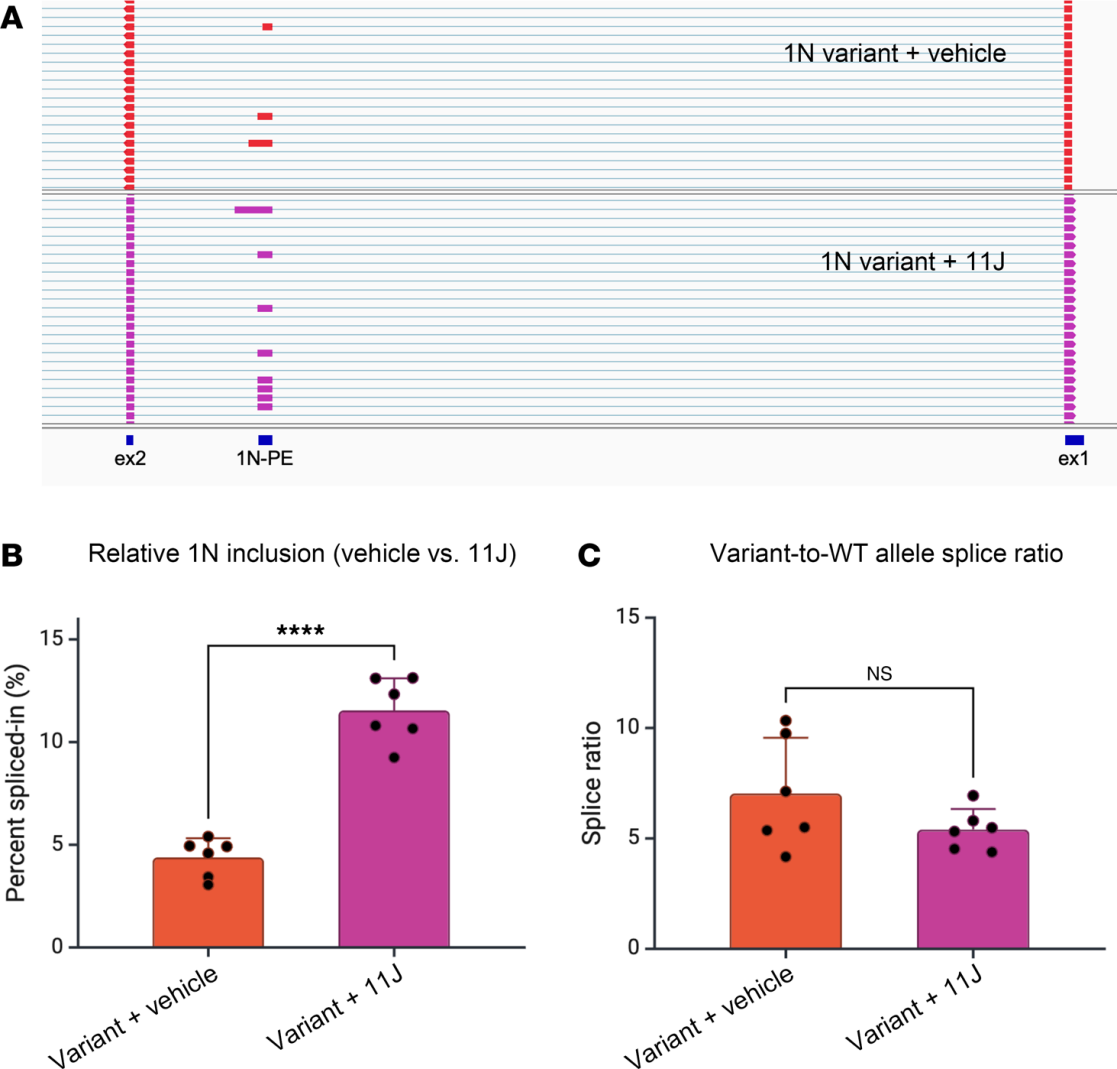
1N inclusion in patient iNeurons is sensitive to inhibition of nonsense-mediated decay (NMD). (**A**) Sample reads from targeted RT-PCR and long-read sequencing of *SCN1A* transcripts in 1N variant–patient iNeurons treated with vehicle or the NMD inhibitor 11J. PE, poison exon. (**B**) Increased relative inclusion of 1N in 11J-treated compared to vehicle-treated patient iNeurons (*n* = 6 biological replicate wells of iNeurons per condition). iNeurons were treated for 24 hours with 1 μM 11J. (**C**) Similar variant-to-WT allele splice ratio in vehicle- or 11J-treated patient iNeurons (*n* = 6 biological replicate wells of iNeurons per condition). ****P < 0.0001; NS, not significant by unpaired *t* test (**B** and **C**)

Given that many genes demonstrate neuron-specific splicing patterns, we next investigated whether a poison-exon-related variant impacts splicing in a neuronal context. Using reprogrammed fibroblasts from a patient with Dravet syndrome carrying a 1N-related variant, we generated iNeurons using a well-described protocol (18). For comparison, we also generated iNeurons from 3 distinct iPSC lines derived from healthy controls. In each of the control iNeuron samples, less than 1% of reads from targeted long-read sequencing contained the poison exon. In contrast, in iNeurons carrying the 1N-related variant, 1N inclusion increased 16-fold, on average, to 3%–6% (Figure 4, A and B, and Supplemental Figure 5). Importantly, long-read sequencing allows the phasing of the transcripts from wild-type (WT) or variant alleles in the heterozygous iNeurons. We calculated the ratio of 1N-containing reads derived from the variant or WT allele. When this ratio significantly deviates from 1, it suggests an allelic imbalance where transcripts are derived preferentially from one allele over the other. In patient iNeurons, we found that this ratio was significantly greater than 1 (Figure 4, C and D), suggesting that 1N-containing reads are preferentially derived from the allele carrying the pathogenic variant. Interestingly, the length of the 1N poison exon is also polymorphic in patient iNeurons, with reads showing multiple alternative 3′ donor splice sites (Supplemental Figure 6). Of note, all the isoforms of 1N are predicted to introduce a PTC when spliced into the *SCN1A* transcript. Given that PTC-containing transcripts are expected to undergo NMD, we investigated the sensitivity of 1N-containing *SCN1A* transcripts to the NMD inhibitor 11J. We found that the inhibition of NMD led to a further 2.6-fold increase in relative 1N inclusion in patient-derived iNeurons without a significant change in allelic imbalance (Figure 5, A–C). Taken together, these data suggest that aberrant poison exon inclusion contributes to Dravet syndrome through unproductive splicing and targeting of *SCN1A* transcripts for NMD.

**Figure 6:**
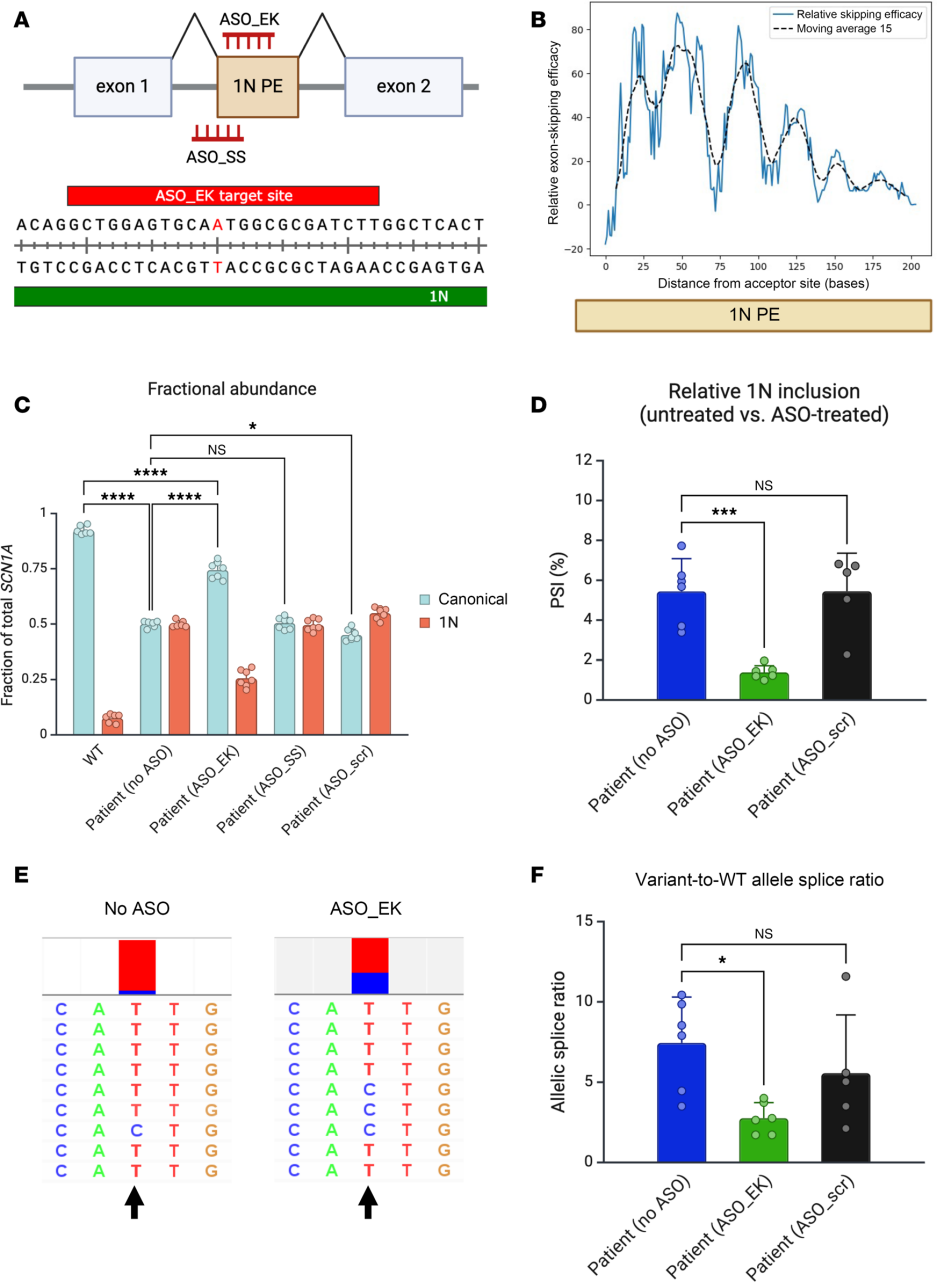
An exon-skipping ASO ameliorates aberrant exon inclusion in patient iNeurons and shows a substantial degree of allele specificity. (**A**) ASO design for targeting ASOs. The ASO_EK (exon-skipping) targeting site lies entirely within the 1N poison exon and overlaps with the 1N patient variant, and therefore has a 1-nucleotide mismatch with the WT allele. ASO_SS (splice-switching) targets the splice acceptor site with the 3′ end of the upstream adjacent intron. A scrambled version of ASO_SS was used as an additional control. PE, poison exon. Created with BioRender. (**B**) eSkip-Finder predictions showing increased likelihood of exon-skipping for targets closer to the acceptor splice site at the 5′ end of 1N. ASO_EK overlaps with positions 25–49 of 1N. (**C**) ddPCR shows that in WT iNeurons, 1N-containing transcripts account for less than 10% of total *SCN1A* transcripts. In contrast, patient iNeurons show similar proportions of canonical or 1N-containing transcripts. ASO_EK significantly shifted the relative abundance of canonical and 1N-containing transcripts in favor of the canonical transcript. In contrast, untreated iNeurons and ASO_SS had similar ratios of canonical and 1N-containing transcripts. The scrambled ASO shifted the relative abundance of canonical and 1N-containing transcripts slightly toward 1N (*n* = 6–7 biological replicate wells of iNeurons per condition). In the patient (no ASO) condition, 1 sample was excluded due a significant result on Grubb’s outlier test (*P* < 0.05). (**D**) Targeted RT-PCR and long-read sequencing was used to assess relative inclusion of 1N in untreated patient iNeurons or those treated with ASOs; ASO_EK, but not ASO_scr, improved the aberrant inclusion of 1N (*n* = 5–6 biological replicate wells of patient iNeurons per condition). (**E**) Representative set of reads containing 1N in untreated iNeurons or iNeurons treated with ASO_EK. Position of the pathogenic variant is indicated by an arrow, with the color map above showing the relative proportion of reads containing T (patient variant, in red) versus C (WT variant, in blue). Reads from untreated iNeurons are strongly skewed toward the variant allele. In contrast, reads from ASO_EK-treated iNeurons show reduced skewing toward the variant allele. (**F**) ASO_EK significantly decreases the variant-to-WT allele splice ratio in patient iNeurons, suggesting that it has a substantial degree of allele-specific action (*n* = 5–6 biological replicate wells of patient iNeurons per condition). *P < 0.05; ***P < 0.001; ****P < 0.0001 by 1-way ANOVA with Dunnett’s multiple-comparison test. NS, not significant.

We next asked whether rationally designed ASOs could rescue the aberrant inclusion of 1N in patient iNeurons. We designed 2 ASOs, targeting either the 5′ splice junction or a possible exon-skipping sequence within 1N (Figure 6A), as well as a scrambled ASO. For the exon-skipping ASO, we used a tool (eSkip-Finder) to assist with target site selection, based on a machine-learning approach to maximize the likelihood of exon-skipping action for ASOs (19). eSkip-Finder showed that for 1N, the highest likelihood of exon skipping occurred for ASOs targeting the 5′ end (Figure 6B). We therefore designed this ASO to overlap with positions 25–49 of 1N, which maximizes the exon-skipping probability and ensures targeting of all alternatively spliced 1N isoforms. The exon-skipping ASO also overlaps with the patient variant, and we selected the nucleotide that matches the disease variant to attempt to gain a degree of allele specificity (Figure 6A). Additionally, we designed a splice-switching ASO targeting the acceptor splice site at the 5′ end of 1N. We did not attempt to design an ASO targeting the numerous donor splice sites at the 3′ end of 1N (Supplemental Figure 7). We tested the efficacy of the 2 ASOs through gymnotic application to iNeurons over 4 days, followed by quantification of *SCN1A* transcript abundance through digital droplet PCR (ddPCR), using primers and probes specific for *SCN1A* transcripts including or excluding the 1N poison exon. In control iNeurons, we found that less than 10% of *SCN1A* transcripts contained the 1N poison exon (Figure 6C). In untreated patient iNeurons, the relative abundances of canonical and 1N-containing transcripts were similar (approximately 50% each), supporting the increased inclusion of 1N due to the patient variant (Figure 6C and Supplemental Figure 7). Next, we found that iNeurons treated with the exon-skipping ASO had a significantly reduced fractional abundance of 1N-containing *SCN1A* transcripts and conversely, increased fractional abundance of the canonical *SCN1A* transcripts (Figure 6C). Total *SCN1A* transcript abundance, estimated as a sum of canonical and 1N-containing transcripts, was overall lower in patient iNeurons compared with control iNeurons, but not significantly different across ASO-treated and untreated samples (Supplemental Figure 7D). The ASO intended to cause splice switching through the targeting of the 5′ splice junction, however, appeared to be ineffective in modulating the relative abundance of canonical and 1N-containing transcripts. Interestingly, the scrambled ASO led to a small but significant effect of increasing relative 1N inclusion. Given that its sequence does not target *SCN1A* or known interacting genes, this may represent an adverse off-target effect or a nonspecific effect on NMD that overall favors poison exon inclusion.

As a complementary approach to assessing poison exon inclusion, we carried out targeted long-read sequencing of *SCN1A* transcripts in ASO-treated iNeurons, which confirmed a reduction in relative 1N inclusion in patient iNeurons treated with the exon-skipping ASO compared with untreated iNeurons (Figure 6D). Additionally, we also examined the effect of the ASOs on the allelic imbalance observed in patient iNeurons. Interestingly, we found that the variant-to-WT allelic splice ratio was significantly reduced by the exon-skipping ASO but not the scrambled ASO. This suggests that the exon-skipping ASO partially corrected the allelic imbalance in patient iNeurons, suggesting a substantial degree of allele specificity for the disease-variant-carrying allele (Figure 6, E and F), a remarkable finding given that there is only a single nucleotide mismatch between the targeting ASO and WT allele. Taken together, our results demonstrate that rationally designed ASOs can ameliorate 1N-mediated pathologic splice patterns and achieve a degree of allele specificity based on minimal sequence mismatch.

## Discussion

### Aberrant inclusion of poison exons as a pathogenic mechanism for deep intronic SCN1A variants.

In this study, we combined targeted RT-PCR with deep-coverage long-read sequencing to reveal 2 poison exons in *SCN1A*, termed 1N and 22N, and demonstrate their inclusion across a range of neural cells and tissues, including the developing human fetal brain. We show that Dravet syndrome–related variants in 1N and 22N increase the aberrant inclusion of their respective poison exons, in vitro and in iNeurons. Of note, we used 2 complementary approaches, ddPCR and targeted RT-PCR with long-read sequencing. ddPCR provides absolute quantification of transcript abundance, whereas targeted RT-PCR with long-read sequencing carries the advantage of being able to phase reads to either the WT or disease variant allele. The latter technique, however, is subject to PCR amplicon length bias and therefore tends to underestimate poison exon inclusion, because poison-exon-containing amplicons are longer than their canonical counterparts. Therefore, the percentage spliced-in (PSI) values in this study should be considered estimates rather than absolute measurements. Using these approaches, we show in the presence of Dravet-related 1N and 22N variants, these poison exons are aberrantly spliced in a disease-relevant context, supporting a pathogenic mechanism of *SCN1A* deficiency. Future studies should aim to clarify the effects of poison-exon-associated variants on Na_V_1.1 protein levels, electrophysiologic function (20), and Dravet-related disease phenotypes. To achieve these goals, substantial technical barriers need to be overcome, which include (a) the low level of Na_V_1.1 in iNeurons due to their limited maturity despite prolonged time in culture, particularly regarding their splicing profiles (21); (b) the challenges in accurate quantification of this protein in Western blotting in multiple contexts (20, 22–24); and (c) the poor sequence conservation of 1N and 22N that precludes the generation of rodent models that recapitulate the genetic context of variants associated with these poison exons. Despite these limitations, previous studies in the 20N poison exon have shown good correlation between poison exon inclusion in vitro, Na_V_1.1 protein levels, and disease phenotypes in rodent models (7, 8, 12), suggesting that stem cell–derived neurons can continue to serve as a useful model for preclinical studies.

The discovery of multiple *SCN1A* poison exons raises questions about their physiologic functions. 20N, which is included in *SCN1A* transcripts at a high level prenatally and downregulated in the postnatal period, appears to serve an important regulatory role in upregulating Na_V_1.1 protein during neuronal maturation. In contrast, the low inclusion rates of 1N and 22N do not suggest a clear physiologic role outside of a disease context, although we cannot rule out the possibility that these poison exons may serve key roles in specific cell types or developmental stages that have not yet been studied. Finally, it remains unknown whether *SCN1A* poison exons may be co-regulated, and how targeting one poison exon may affect the others.

Of note, the 1N poison exon lies within a SINE, a type of transposable element (TE). This is an interesting finding that reveals the disease relevance of TEs. Emerging evidence shows that TEs can serve unique roles in gene regulation (25). When inserted into an intronic region, TEs can undergo a process called exonization, generating chimeric transcripts through alternative splicing. As in the case of the 1N poison exon, exonized TEs often contain multiple alternative donor or acceptor splice sites (26). Although these exonized TEs are usually spliced in at low rates, disease variants can dramatically alter their splicing and lead to deleterious effects on gene expression. Poison exons derived from TEs, therefore, may serve an underappreciated role in gene regulation and represent an important source of pathogenic noncoding variation in the genome at large.

Interestingly, both the 1N- and 22N-related variants in this study lie within their respective poison exons. This suggests that they may disrupt specific splice-modulatory motifs. For example, the 1N variant may generate a de novo exonic splice enhancer that may be sterically blocked by the exon-skipping ASO tested in this study. Another potential mechanism may involve the disruption of binding sites for hnRNPA1, a splice silencer (9). Further studies are needed to identify the mechanisms by which regulatory motifs within poison exons may impact their interactions with RNA-binding proteins in physiologic and disease contexts.

### ASO modulation of splicing and therapeutic potential.

In this study, we leverage the unique potential of ASO technology and show that they can ameliorate aberrant patterns of poison exon inclusion in a disease context. Of note, all ASOs used in this study were fully modified with 2′-MOE, to facilitate a steric-blocking mechanism and avoid RNase H–mediated degradation of the target transcript. Using *SCN1A* 1N and 22N, we show that ASOs targeting either the splice junctions of poison exons or internal sequences have the potential to ameliorate aberrant exon inclusion. Remarkably, we also show that a single nucleotide mismatch from the WT allele allows an ASO targeting 1N to gain a substantial degree of allele-specific action. Taken together, our results highlight the opportunity for precision medicine in patients carrying poison-exon-related variants. Furthermore, 1N and 22N could represent therapeutic targets to fine-tune Na_V_1.1 protein abundance across the spectrum of *SCN1A*-related disorders.

### Developmental regulation of SCN1A poison exons.

Several gene therapy approaches are currently being evaluated for *SCN1A*-related Dravet syndrome, including an ASO targeting unproductive splicing (8) and an engineered transcription factor (27). These strategies share the common aim of increasing the total amount of functional *SCN1A* transcripts to rescue an *SCN1A*-deficient disease state. Poison exons, similarly, leverage an endogenous regulatory mechanism of protein abundance. Therefore, knowledge of the developmental regulation of poison exons is critical to understanding the potential opportunities as well as barriers for efficacious gene therapy. In this study, we directly examine, for the first time to our knowledge, *SCN1A* poison exon inclusion in human brain tissue across prenatal and postnatal development, highlighting the developmental downregulation of 20N in human brain tissue. Interestingly, our data indicate that 20N remains at high levels in the early postnatal period, suggesting the possibility of an extended critical window for ASO therapy for Dravet syndrome during childhood. Finally, although *SCN1A* is known to be highly expressed across multiple neuronal cell types in the human brain (28), it is unclear whether it is differentially spliced across these distinct cell types. Future studies, therefore, should investigate *SCN1A* poison exon inclusion across development and distinct cell types, particularly during early postnatal years, to determine the ideal time window and spatial context for modulation of *SCN1A* transcript expression and splicing.

## Methods

### Sex as a biological variable.

The 2 patients whose variants were studied were female. Both male and female control iPSC cell lines and fetal brain samples were used. The sexes of the adult individuals from whom brain RNA was generated were unavailable.

### iPSC generation and quality control.

Three control and 1 patient iPSC line were used in this study. Two control lines were purchased as fibroblasts: GM03651 (Coriell Institute, apparently healthy female, 25 years old at sampling) and GM03552 (Coriell Institute, apparently healthy male at 24 years old at sampling). One control iPSC line (H11, male) was generated as part of a previous study and a gift from the University of Michigan (29). For all experiments involving control iPSC-derived neurons, an equal sample size from each of 3 control lines (*n* = 2 per line, for a total of 6 biological replicate wells) was used. Control and patient fibroblasts were reprogrammed by the Northwestern University Stem Cell Core using a CytoTune-iPS 2.0 Sendai Reprogramming Kit (Thermo Fisher Scientific, A16517) and assessed for pluripotency markers. We confirmed using Sanger sequencing that the patient iPSC line is heterozygous for the expected 1N-related variant (Supplemental Figure 3). All iPSC lines were tested using the PCR-based Venor GeM Mycoplasma Detection Kit (MilliporeSigma, MP0025) and verified to be free of mycoplasma contamination.

For assessment of genomic integrity, whole-genome sequencing was conducted for all iPSC lines used in this study. Briefly, genomic DNA was prepared using a PureLink Genomic DNA Mini Kit (Thermo Fisher Scientific, K182002) and submitted for whole-genome sequencing at Novogene using the Illumina NovaSeq X Plus/NovaSeq 6000 at 30× coverage with 150-bp paired-end reads. Raw reads were aligned to the genome using SpeedSeq (30). Structural variants (SVs) were called by 3 independent SV callers, Lumpy (31), Manta (32), and CNVnator (33). SV calls were genotyped and integrated into a single variant call format (VCF) file by the consensus caller MetaSV (34). The VCF was then parsed with a custom Python script into BED format (supplemental material). Using size inclusion criteria from a previous study (35), we filtered SVs based on size (>100,000 kb). Custom annotation was performed with BEDTools (36) to annotate the overlap with SVs found in the general population (gnomAD SV) (37). We identified no coding SVs that were absent from gnomAD SV in any of our control or patient iPSC lines.

### iPSC culture and neuronal differentiation.

iPSCs were maintained in mTeSR+ (STEMCELL Technologies, 1000276) on plates coated with Matrigel (Corning, 354277). Each iPSC line was transduced with 2 lentiviral vectors, tetO-*Ngn2*-eGFP (gift from Scott Adney lab, Northwestern University) and CAG-rtTA (University of Iowa Viral Vector Core, 2467), to generate iPSC lines with doxycycline-inducible neurogenin 2 (*Ngn2*) expression. Neuronal differentiation was conducted through a well-established protocol (18). Briefly, on differentiation day 0 (DD0), doxycycline (2 μg/mL; Thermo Fisher Scientific, J67043-AD) was used to induce differentiation, followed by selection with puromycin (5 μg/mL, Thermo Fisher Scientific, J67236-XF) for 48 hours. On DD3, differentiating iNeurons were replated onto 6-well plates coated with double-concentration Matrigel, at a density of 750,000 cells per well, in neuronal maintenance media containing Neurobasal medium (Thermo Fisher Scientific, 21103049), GlutaMax (Thermo Fisher Scientific, 35050061), MEM NEAA (Thermo Fisher Scientific, 11140050), GEM 21 (Gemini Bio, 400161010), N2 (Gemini Bio, 400163005), BDNF (10 ng/mL; PeproTech, 450-02), NT3 (10 ng/mL; PeproTech, 450-03), and laminin (200 ng/mL; MilliporeSigma, 11243217001). Thereafter, a 50% media exchange was performed on DD5, DD7, and DD10. Ara-C (2 μM; MilliporeSigma, C1768) was added to the media from DD5 to DD7. Doxycycline was discontinued on DD10. Specifically, no glia were added to the differentiation protocol to reduce the possibility of contaminating reads from glial *SCN1A* transcripts. All iNeurons were harvested on DD14 for total RNA extraction. Compared with control iNeurons, iNeurons generated from the patient carrying the 1N-related variant had grossly normal appearing morphology and survival during differentiation (Supplemental Figure 4).

For the inhibition of NMD, 11J or hSMG-1 inhibitor (MedChemExpress, HY-124719) was dissolved in DMSO at 1 mM to make a stock solution. The stock solution was diluted in media for a final working concentration of 1 μM. iNeurons were treated for 4–24 hours starting on DD13 and were collected for RNA extraction on DD14.

### Fetal and adult human brain tissue and RNA.

Fetal human brain tissues (13, 15, and 18 postconception weeks [pcw]) was provided by the Birth Defects Research Laboratory (BDRL) at the University of Washington.

Fetal human brain tissue (23 pcw) was shared with us by the Richard Smith lab (Northwestern University). Fetal brain tissue was received after release from clinical pathology, with a maximum postmortem interval of 4 hours. Fetal cases with known anomalies were excluded. Tissue was transported in ice-cold Hibernate-E medium (Thermo Fisher Scientific, A1247601) for processing in the laboratory. Postnatal human brain samples were obtained from the University of Maryland Brain and Tissue Bank of the NIH NeuroBioBank and Miami Biobank, with the sample numbers UMBN4353 (1 month old) and HCT17HEIA029 (3 years old).

Fetal and early postnatal tissues were dissected to remove cortical regions that were homogenized in TRIzol (Thermo Fisher Scientific, 15596026) for RNA extraction with the standard protocol described above for iNeurons, followed by Zymo RNA Clean & Concentrate Kit 5 (Zymo Research, 4004). Two adult human brain total RNA samples were used in this study, both purchased commercially: adult 1 refers to a sample of human cerebral cortex total RNA, pooled from 3 individuals aged 24–27 years (Takara Bio, 636561, lot 2407059); adult 2 refers to a sample of human cerebral cortex total RNA from a single 27-year-old individual (BioChain, R1234042-10, lot B810027).

### Targeted SCN1A RT-PCR and long-read sequencing.

Total RNA from iNeurons was extracted using a standard TRIzol-chloroform protocol followed by the Zymo RNA Clean & Concentrate Kit 5 (Zymo Research, 4004). One microgram of each RNA sample was used for reverse transcription with Maxima H Minus cDNA synthesis master mix (Thermo Fisher Scientific, M1662). The resulting cDNA was used for targeted PCR with *SCN1A-*specific primers (Supplemental Table 2). The PCR product was purified using a QIAquick PCR purification kit (Qiagen, 28104). Samples were sent to Plasmidsaurus for long-read sequence using the Premium PCR service. At least 1000 high-quality reads mapping specifically to *SCN1A* were obtained per sample. Reads were mapped using minimap2 (38), and reads covering or including the poison exon were extracted using the intersect function from BEDTools, using an approach described in Supplemental Figure 1. The PSI was calculated as the proportion of reads containing at least a part of the poison exon out of the total number of reads that completely covered the target region. Variant-to-WT allele ratio was calculated using the reads that contained the poison exon of interest. Reads were visualized in Integrative Genomics Viewer (https://igv.org/).

### 22N splice reporter assay generation.

The *SCN1A* 22N splice reporter assay was generated using a similar approach as described previously (4, 6). A minigene containing human *SCN1A* exons 22 and 23 with the entire intervening intronic sequence, as well as flanking intronic regions (500 bp upstream and downstream of the adjacent exons), was synthesized as a GeneArt string (Thermo Fisher Scientific) and inserted into pDESTsplice (Addgene, 32484). The splice reporter construct, after insertion into pDESTsplice, is additionally flanked by rat insulin exons, which serve as a constitutively spliced positive control. To introduce the 22N-related patient variant, we conducted site-directed mutagenesis using PCR-based amplification of the control construct with Platinum SuperFi II DNA Polymerase (Thermo Fisher Scientific, 12361010) and mutagenic primers. The PCR product was transformed and circularized into One Shot TOP10 competent *E*. *coli* (Thermo Fisher Scientific, C404006). Single colonies were screened for the presence of the 22N-related variant. The size of the final control and 22N variant constructs were verified with restriction digest and agarose gel electrophoresis. Long-read sequencing of each construct was conducted to verify that the control and 22N variant constructs differed only at the position of the patient variant.

### HEK293T cell line culture and transfections.

HEK293T cells were cultured using DMEM supplemented with 10% (v/v) fetal bovine serum and 1% (v/v) penicillin-streptomycin in 6-well plates. For the 22N splice reporter assay, HEK293T cells were transfected with 5 μg of control or variant splice reporter construct using TurboFectin 8.0 (OriGene). Cells were collected in TRIzol reagent 24 hours after transfection, and total RNA was extracted using a standard TRIzol-chloroform protocol followed by the Zymo RNA Clean & Concentrate Kit 25 (Zymo Research, 4033). For screening of ASOs targeting *SCN1A* 22N, HEK293T cells were additionally transfected with each ASO (or no ASO) using TurboFectin 8.0 (OriGene) at a concentration of 1 μM. Total RNA was collected 24 hours after ASO transfection and used for quantitative RT-PCR (qRT-PCR).

### qRT-PCR.

Total RNA from HEK293T cells was analyzed by qRT-PCR with an iTaq Universal SYBR Green One-Step Kit (Bio-Rad, 1725150), using primers specific for the canonical or 22N-containing splice products from the 22N splice reporter assay. Primers specific for the constitutive rat insulin splice product generated by the splice reporter assay were used as a control, which normalizes for differences in transfection efficiency and differences in splice reporter expression. Three technical replicate reactions, each using 200 ng of RNA, were conducted for each biological replicate. Ct values were calculated for each *SCN1A* splice product (canonical or 22N-containing) or the constitutive insulin splice product; ΔCt was calculated by subtracting the Ct for each *SCN1A* splice product by the Ct for the constitutive insulin splice product, or by subtracting the Ct of the canonical *SCN1A* splice product by the Ct of the 22N-containing splice product.

### ASO design.

We followed existing guidelines for the design of splice-switching and exon-skipping ASOs (39), with the following features: GC content 40%–60%, 18–22 nucleotide in length, and avoiding 3 G nucleotides in a row. BLAST was used to avoid greater than 17 consecutive nucleotides of homology with any off-target sequence, particularly with respect to other sodium ion channels. Splice-switching ASOs were designed with the sequence partially overlapping the exon-intron junction. ASO sequences with their chemical modifications are shown in Supplemental Table 1. All ASOs were made with full phosphorothioate linkages and 2′-MOE modifications throughout, which confer resistance to RNase H degradation and facilitate the desired steric blocking mechanism. For the exon-skipping ASO for 1N, eSkip-Finder was used to find a target at the 5′ end of 1N with high probability of exon-skipping. Information for all ASOs used in this study are displayed in Supplemental Table 1.

### ASO administration in iNeurons.

ASOs were administered to iNeurons from the patient carrying the 1N-related variant. Specifically, each ASO was applied via gymnosis (in the absence of any transfection reagents or carrier molecules, by directly diluting ASO stock solution into media) at 10 μM final concentration, as part of a 50% media exchange on DD10 at 10 μM through DD14, for a total of 96 hours, prior to collection in TRIzol reagent for total RNA extraction.

### ddPCR.

Total RNA from iNeurons was extracted using a standard TRIzol-chloroform protocol followed by the Zymo RNA Clean & Concentrate Kit 5 (Zymo Research, 4004). One microgram of each RNA sample was used for reverse transcription with Maxima H Minus cDNA synthesis master mix (Thermo Fisher Scientific, M1662). RNA-equivalent cDNA (125 ng) was used for each ddPCR reaction, which was set up using the ddPCR Supermix for Probes (no dUTP) (Bio-Rad, 1863023). For each reaction, 70 μL of Droplet Generation Oil for Probes (Bio-Rad, 1863005) and 20 μL of the PCR reaction was loaded into a DG8 cartridge (Bio-Rad, 1864008). Droplets were generated in a QX200 Droplet Generator and subsequently transferred to a 96-well plate for PCR, followed by analysis on the QX200 Droplet Reader. Data were collected and amplitude thresholds and concentrations (in copies/μL) were automatically set by the QX Manager Software Suite. For the *SCN1A* canonical and 1N-containing transcripts, a common probe (PrimeTime probe, Integrated DNA Technologies) targeting canonical exon 1 was used, with specific primer sets targeting the unique exon-exon junctions for each splice isoform. Each biological replicate resulted in 3 separate ddPCR reactions, each with technical replicates, resulting in 6 total ddPCR reactions per sample. The 3 separate ddPCR reactions consisted of (a) a custom primer/probe set targeting the *SCN1A* canonical transcript (with FAM fluorophore), (b) a custom primer/probe set targeting the *SCN1A* 1N-containing transcript (with FAM fluorophore), and (c) a commercial assay for human TATA-binding protein (*TBP*) (Thermo Fisher Scientific, assay ID Hs00608272_m1, with VIC fluorophore in primer-limited formulation).

### Primer and probe design for targeted RT-PCR, qRT-PCR, and ddPCR.

All primers were designed with Primer3 with the following parameters: GC content 40%–60%, target T_m_ 60°C, and avoiding repeats of G or C nucleotides longer than 3 bases. An extended primer with lower GC content was designed for the *SCN1A* exon 1–exon 2 junction to ensure specificity for *SCN1A*. Primers were additionally checked for self-complementarity and by BLAST (https://blast.ncbi.nlm.nih.gov/Blast.cgi) to ensure target specificity, and conventional endpoint PCR was conducted to ensure the presence of a single band at the expected size. Of note, all primers targeting *SCN1A* were checked to ensure a high degree of mismatch (at least 25% of the sequence) with other sodium channel genes given their high degree of homology. Information for all RT-PCR and ddPCR primers and probes used in this study are displayed in Supplemental Table 2.

### Statistics.

For the splice reporter assay and ASO experiments in HEK239T cells, a power analysis was performed based on previous studies involving the *SCN1A* poison exon 20N (4, 8), and similar sample sizes were used for our experiments. For targeted long-read sequencing experiments in iNeurons, we first performed a pilot experiment with a sample size of *n* = 3 per genotype and replicated the experiment a second time with an additional *n* = 3 per genotype. Based on the effect size obtained from this experiment, we used similar sample sizes for all other experiments in iNeurons, including 11J treatment and ASO testing.

All data summary graphs were created in BioRender. Statistical analysis was done directly in BioRender. All *t* tests were 2-tailed. For specific tests not available in BioRender (i.e., Shapiro-Wilk test and 1-sample *t* test), GraphPad Prism or R was used. All datasets were first assessed for deviation from normality using the Shapiro-Wilk test. Datasets that deviated significantly from a normal distribution were analyzed using nonparametric methods. For pairwise comparisons, an unpaired *t* test was used for parametric data, and a Mann-Whitney test was used for nonparametric data. For datasets with multiple comparisons, 1-way ANOVA was used for parametric data, and the Kruskal-Wallis test was used for nonparametric data. Dunnett’s multiple-comparison test was used for post hoc comparisons, where each ASO-treated sample was compared to the untreated condition. For datasets involving ASO-treated iNeurons, due to the variability observed for the abundance of *SCN1A* transcript isoforms, Grubb’s outlier test was applied to all samples. Error bars represent 1 standard deviation (SD), unless otherwise stated. A *P* value of less than 0.05 was considered significant. Significant outliers (*P* < 0.05) were removed from all analyses; overall, this resulted in removal of 1 biological replicate from the untreated condition.

### Study approval.

Stem cell research was performed in compliance with relevant ethical regulations and approved by the Northwestern University Institutional Review Board as nonhuman subject research (STU00204022). For the patient iPSC line, fibroblasts were obtained from the patient carrying the 1N variant (Table 1) after written informed consent was obtained from parents/legal guardians. All fetal human brain work was performed according to guidelines for the research use of human brain tissue at Northwestern University with an exemption due to a “Not Human Subjects Research” determination by the Institutional Review Board (STU00219975).

### Data availability.

The custom Python script is available in the supplemental material. Values for all data points in graphs are reported in the Supporting Data Values file. Raw data for all targeted long-read sequencing datasets have been deposited in the NCBI Sequence Read Archive (SRA) under the accession PRJNA1221960.

## Author contributions

ST designed the experiments and reviewed and analyzed the data. HS and SW facilitated the genetic diagnosis and collection of fibroblasts for the patient carrying the 1N variant. GLC and JDC assisted with experimental design and data interpretation. ST wrote the manuscript with input from all the other authors.

## Supplementary Material

Supplemental data

Supporting data values

## Figures and Tables

**Table 1:**
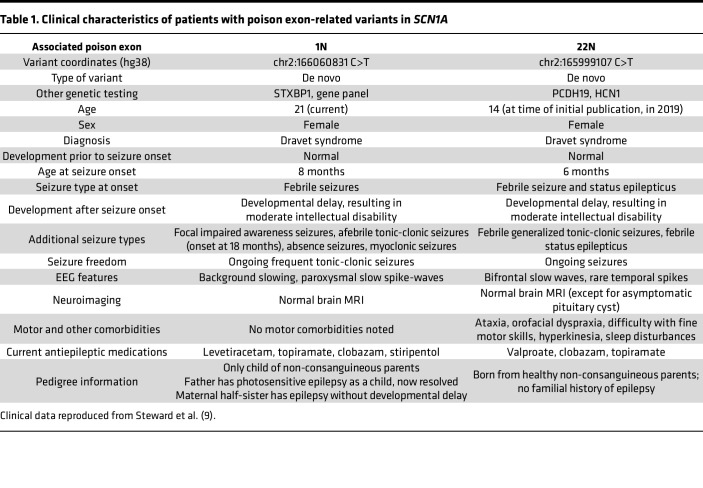
Clinical characteristics of patients with poison exon-related variants in *SCN1A*

**Table 2:**
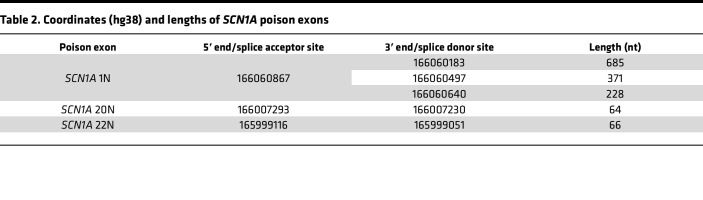
Coordinates (hg38) and lengths of *SCN1A* poison exons

## Version 1 02/13/2025

In-Press Preview

## Version 2 04/08/2025

Electronic publication

## References

[1] Happ HC, Carvill GL (2020). A 2020 view on the genetics of developmental and epileptic encephalopathies. Epilepsy Curr.

[2] Scheffer IE, Nabbout R (2019). SCN1A-related phenotypes: epilepsy and beyond. Epilepsia.

[3] Dravet C (2011). The core Dravet syndrome phenotype. Epilepsia.

[4] Carvill GL (2018). Aberrant inclusion of a poison exon causes dravet syndrome and related SCN1A-associated genetic epilepsies. Am J Hum Genet.

[5] Carvill GL, Mefford HC (2020). Poison exons in neurodevelopment and disease. Curr Opin Genet Dev.

[7] Han Z (2020). Antisense oligonucleotides increase Scn1a expression and reduce seizures and SUDEP incidence in a mouse model of Dravet syndrome. Sci Transl Med.

[8] Lim KH (2020). Antisense oligonucleotide modulation of non-productive alternative splicing upregulates gene expression. Nat Commun.

[9] Steward CA (2019). Re-annotation of 191 developmental and epileptic encephalopathy-associated genes unmasks de novo variants in *SCN1A*. NPJ Genom Med.

[10] Zhang X (2016). Cell-type-specific alternative splicing governs cell fate in the developing cerebral cortex. Cell.

[11] Yang R (2023). Upregulation of SYNGAP1 expression in mice and human neurons by redirecting alternative splicing. Neuron.

[12] Voskobiynyk Y (2021). Aberrant regulation of a poison exon caused by a non-coding variant in a mouse model of Scn1a-associated epileptic encephalopathy. PLoS Genet.

[13] Peña JB de la (2022). Inhibition of nonsense-mediated decay induces nociceptive sensitization through activation of the integrated stress response. J Neurosci.

[14] Sato H, Singer RH (2021). Cellular variability of nonsense-mediated mRNA decay. Nat Commun.

[15] Dindot SV (2023). An ASO therapy for Angelman syndrome that targets an evolutionarily conserved region at the start of the *UBE3A-AS* transcript. Sci Transl Med.

[16] Chen X (2024). Antisense oligonucleotide therapeutic approach for Timothy syndrome. Nature.

[17] Crooke ST (2021). Antisense technology: a review. J Biol Chem.

[18] Zhang Y (2013). Rapid single-step induction of functional neurons from human pluripotent stem cells. Neuron.

[19] Chiba S (2021). eSkip-Finder: a machine learning-based web application and database to identify the optimal sequences of antisense oligonucleotides for exon skipping. Nucleic Acids Res.

[20] Hugte EJH van (2023). SCN1A-deficient excitatory neuronal networks display mutation-specific phenotypes. Brain.

[21] Weyn-Vanhentenryck SM (2018). Precise temporal regulation of alternative splicing during neural development. Nat Commun.

[22] Cheah CS (2012). Specific deletion of NaV1.1 sodium channels in inhibitory interneurons causes seizures and premature death in a mouse model of Dravet syndrome. Proc Natl Acad Sci U S A.

[23] Verret L (2012). Inhibitory interneuron deficit links altered network activity and cognitive dysfunction in Alzheimer model. Cell.

[24] Wang W (2011). The developmental changes of Na(v)1.1 and Na(v)1.2 expression in the human hippocampus and temporal lobe. Brain Res.

[25] Jönsson ME (2020). Transposable elements: a common feature of neurodevelopmental and neurodegenerative disorders. Trends Genet.

[26] Sorek R (2002). Alu-containing exons are alternatively spliced. Genome Res.

[27] Tanenhaus A (2022). Cell-selective adeno-associated virus-mediated SCN1A gene regulation therapy rescues mortality and seizure phenotypes in a Dravet syndrome mouse model and is well tolerated in nonhuman primates. Hum Gene Ther.

[28] Siletti K (2023). Transcriptomic diversity of cell types across the adult human brain. Science.

[29] Tidball AM (2017). Rapid generation of human genetic loss-of-function iPSC lines by simultaneous reprogramming and gene editing. Stem Cell Reports.

[30] Chiang C (2015). SpeedSeq: ultra-fast personal genome analysis and interpretation. Nat Methods.

[31] Layer RM (2014). LUMPY: a probabilistic framework for structural variant discovery. Genome Biol.

[32] Chen X (2015). Manta: rapid detection of structural variants and indels for germline and cancer sequencing applications. Bioinformatics.

[33] Abyzov A (2011). CNVnator: an approach to discover, genotype, and characterize typical and atypical CNVs from family and population genome sequencing. Genome Res.

[34] Mohiyuddin M (2015). MetaSV: an accurate and integrative structural-variant caller for next generation sequencing. Bioinformatics.

[35] Gracia-Diaz C (2024). KOLF2.1J iPSCs carry CNVs associated with neurodevelopmental disorders. Cell Stem Cell.

[36] Quinlan AR, Hall IM (2010). BEDTools: a flexible suite of utilities for comparing genomic features. Bioinformatics.

[37] Collins RL (2020). A structural variation reference for medical and population genetics. Nature.

[38] Li H (2018). Minimap2: pairwise alignment for nucleotide sequences. Bioinformatics.

[39] Aartsma-Rus A (2023). Consensus guidelines for the design and in vitro preclinical efficacy testing N-of-1 exon skipping antisense oligonucleotides. Nucleic Acid Ther.

[40] Davydov EV (2010). Identifying a high fraction of the human genome to be under selective constraint using GERP++. PLoS Comput Biol.

