# Supplementary material for: Antisense oligonucleotides modulate aberrant inclusion of poison exons in SCN1A-related Dravet syndrome
Source: JCI Insight. 2025 Feb 13;10(7):e188014. doi: 10.1172/jci.insight.188014 (PMC11981616; doi:10.1172/jci.insight.188014)
Supplement: Supplemental data [file jciinsight-10-188014-s149.pdf]

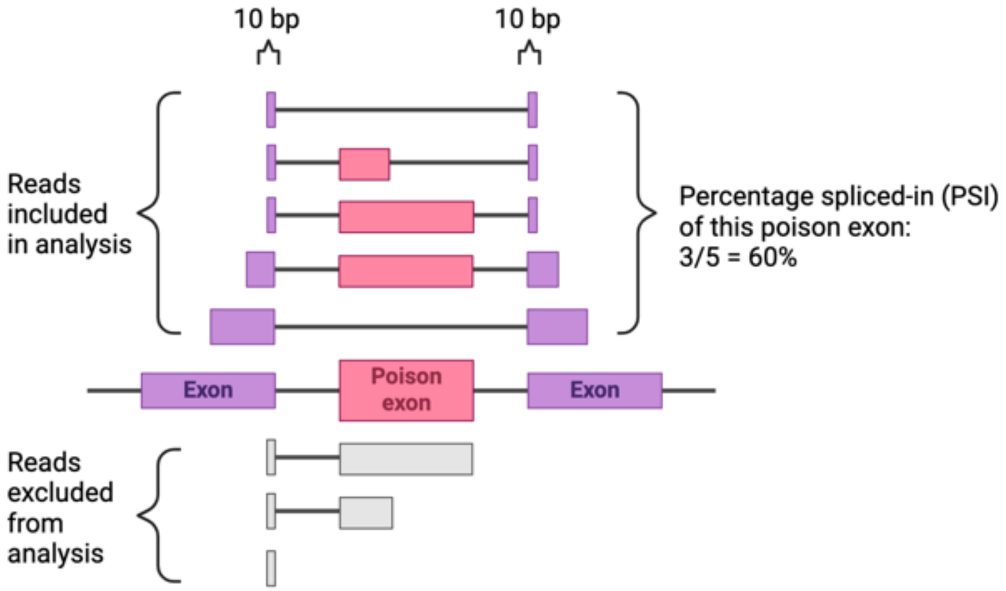

Figure S1. Assessment of percentage spliced-in (PSI) in targeted RT-PCR and long-read sequencing data. Schematic showing the method for assessing PSI for poison exons. For each poison exon, at least 500 high-quality reads mapping to *SCN1A* are first filtered for reads that cover the nearest adjacent constitutively spliced exons with at least a 10-nt overlap. These reads are further filtered for reads that cover any part of the poison exon. PSI is calculated as the percent of reads that include any part of the poison exon region divided by the reads that cover the adjacent canonical exons. This method ensures that reads terminating in the middle of the poison exon and reads covering only one of the flanking canonical exons are not included in the analysis. In the case of 1N, the 228-nt region at the 5' end of the poison exon was designated the poison exon region, which allows for both smaller and larger splice isoforms to be included in the calculation of PSI (Figure S7 shows that essentially all splice isoforms of 1N share the same 5' end sequence). In the case of 22N, exon 22 was used as an adjacent exon rather than exon 21, given alternative splicing of exon 21. Created with BioRender.

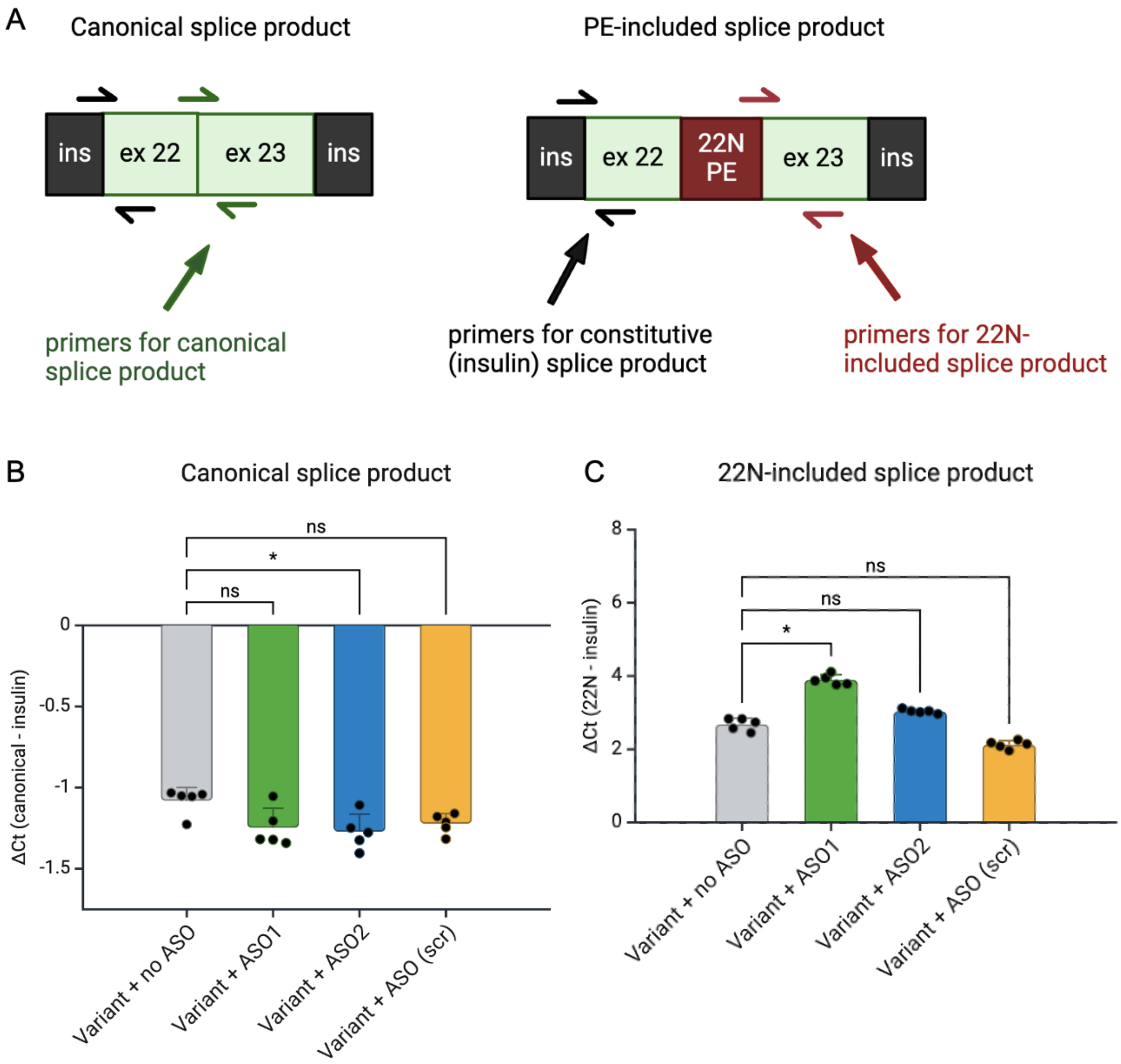

Figure S2. qRT-PCR results for untreated and ASO-treated HEK293T cells expressing a minigene carrying the 22N-related variant.

(A) Locations of primers for canonical or 22N splice products, as well as the constitutive rat insulin splice junction. Created with BioRender.

(B) qRT-PCR result for the canonical splice product, normalized over the constitutively spliced rat insulin exon, displayed as delta Ct values. Kruskal-Wallis test with Dunn's multiple comparisons test, $p = 0.043$; no ASO vs. ASO1, ns ($p = 0.064$), no ASO vs. ASO2, $p = 0.026$; no ASO vs ASO (scr), ns.

(C) qRT-PCR result for the 22N-containing splice product, normalized over the constitutively spliced rat insulin exon, displayed as delta Ct values. Kruskal-Wallis test with Dunn's multiple comparisons test, $p = 0.00047$; no ASO vs. ASO1, $p = 0.022$; no ASO vs ASO2, ns; no ASO vs. ASO (scr), ns.

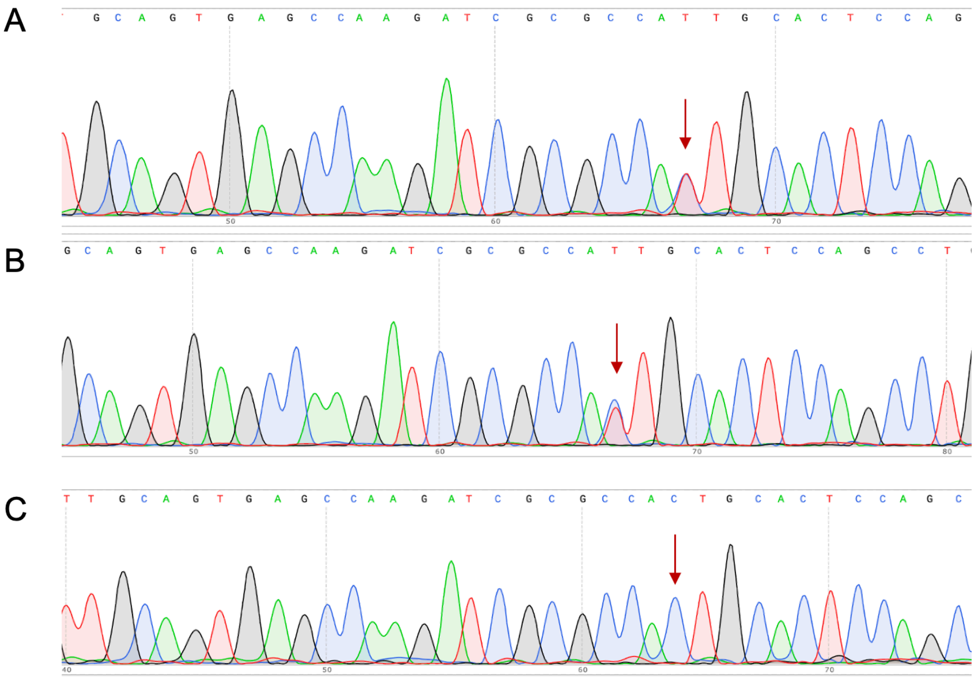

Figure S3. Sanger sequencing confirmation of iPSCs carrying the 1N patient variant.

(A, B) Sanger sequencing traces for a PCR product amplifying the region of the 1N patient variant, using both forward (A) and reverse (B) primers, demonstrating heterozygosity of the chr2:166060831 C>T variant in the patient-derived iPSC line. Sequence at the top of each trace refers to the positive (+) strand of chromosome 2. (C) Sanger sequencing trace of a healthy control iPSC line showing homozygosity of the wild type variant (nucleotide = C).

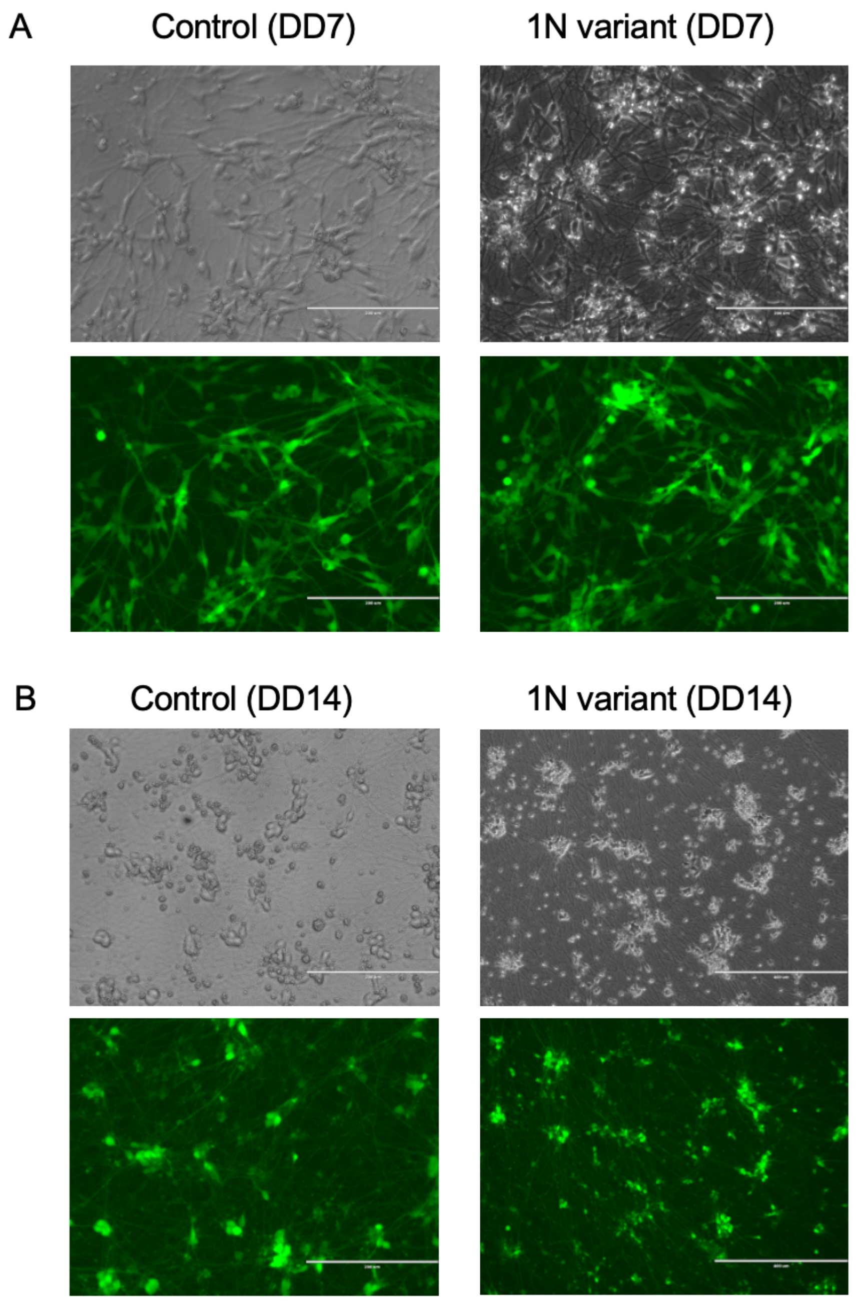

Figure S4. Images of iNeurons from control and patient-derived iPSCs.

(A-B) iNeurons at differentiation day 7 from control (A) and Dravet syndrome patient carrying the 1N-related variant (B). Note that iNeurons were grown in the absence of glia to minimize the contamination of our data from glial *SCN1A* transcripts.

(C-D) iNeurons at differentiation day 14 from control (C) and Dravet syndrome patient carrying the 1N-related variant (D).

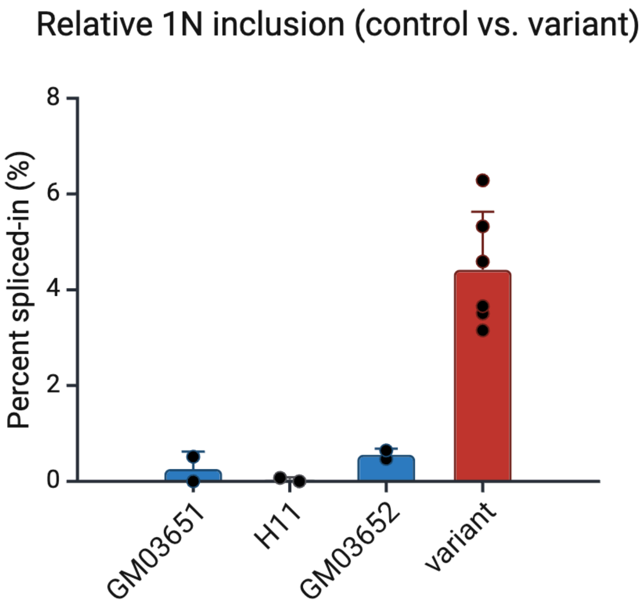

Figure S5. Comparison of relative 1N inclusion in iNeurons derived from control vs. 1N-related variant patient iPSCs.

Data from Figure 3B, shown with separate columns for replicates from distinct control iPSC lines, demonstrating that 1N inclusion was uniformly low across three non-isogenic control iPSC lines compared to the 1N-related patient variant line.

A

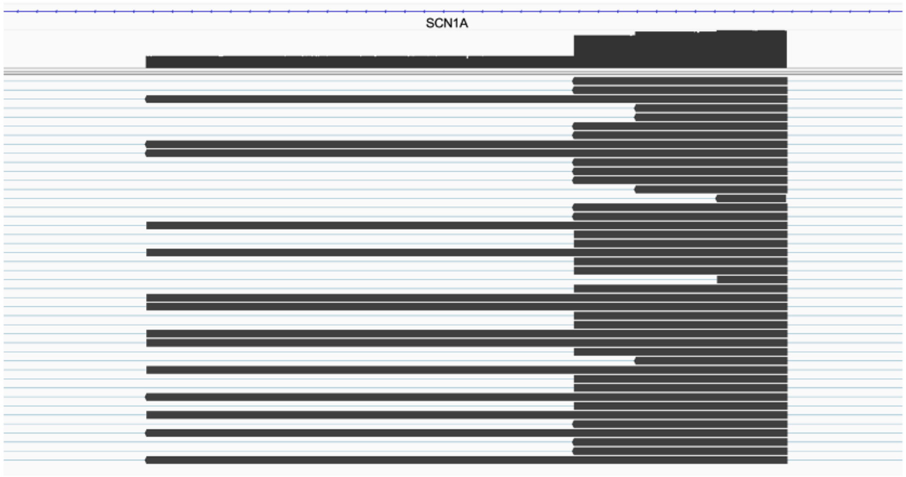

B

Position (bp)	Putative splice site	Sequence	Score*
153	Alt. isoform/cryptic acceptor	tttttttcagATGGAGTTTT	14.737
178	Alt. isoform/cryptic acceptor	catcacacagGCTGGAGTGC	6.364
260	Constitutive acceptor	cctgcctcagCCTCCCAAGC	7.352
269	Alt. isoform/cryptic acceptor	gcctcccaagCAACTGGGAT	2.608
314	Alt. isoform/cryptic donor	TAATTTTTTTgtgtgttttt	7.336
327	Alt. isoform/cryptic acceptor	gtgtttttagTAGAGATGGG	6.314
330	Alt. isoform/cryptic acceptor	tttttagtagAGATGGGGTT	4.721
380	unclassified donor	CTGACCTCAGgtgatgtgcc	8.205
381	Constitutive acceptor	ctgacctcagGTGATGTGCC	6.640
409	Alt. isoform/cryptic acceptor	cctcccaaagTGCTGGGATT	3.188
423	Alt. isoform/cryptic acceptor	gggattacagACGTGAGCCA	2.297
424	Alt. isoform/cryptic donor	GATTACAGACgtgagccact	8.659
471	Alt. isoform/cryptic acceptor	tttctttcagATTTTACTCA	8.265
482	Alt. isoform/cryptic acceptor	ttttactcagCATATCTGGG	3.360
523	Alt. isoform/cryptic donor	CATTTGAAAGgtggaccata	6.771
550	Alt. isoform/cryptic acceptor	cctgagctagCTCACTTTGG	2.564
598	Alt. isoform/cryptic acceptor	tcccccacagCAAAGGCATT	7.010
659	Alt. isoform/cryptic acceptor	tgtcattcagGGACAGAATC	2.820
714	Alt. isoform/cryptic acceptor	gttggcatagGGGATTGTTC	2.596
728	Alt. isoform/cryptic donor	TGTTCTTCTAgtaagcacag	8.405
730	Alt. isoform/cryptic acceptor	gttcttctagTAAGCACAGG	4.504
738	Alt. isoform/cryptic donor	GTAAGCACAGgtgaaattct	10.110
739	Alt. isoform/cryptic acceptor	gtaagcacagGTGAAATTCT	3.696
772	Alt. isoform/cryptic donor	GCGCTTTGTGgtaaagctct	7.077
814	Alt. isoform/cryptic acceptor	tccttcaaagATGATAGAGG	4.366
837	Constitutive donor	GCAGGCAGAGgtgagacttg	13.924

Figure S6. The 1N poison exon is polymorphic and contains multiple alternative donor splice sites.

(A) Coverage map over the 1N poison exon region of reads from targeted RT-PCR and long-read sequencing of 1N-related variant patient iNeurons. Shown are 1N poison exon splice isoforms of various lengths, with the identical acceptor splice site with the 3' end of upstream adjacent intron, but with different donor splice sites with the 5' end of the downstream intron. Across reads from all patient iNeuron samples, at least five different 1N splice isoforms are seen, ranging from 75 to 685 nt (lengths: 75, 162, 228, 371, 685 nt). Each of the five alternative donor splice sites contain the sequence GT. Of note, all isoforms of 1N contain the same PTC.

(B) *In silico* analysis with the Alternative Splice Site Predictor (ASSP) (Wang M, Marín A. Characterization and prediction of alternative splice sites. *Gene*. 2006;366(2):219–227) predicted 4 of 5 observed alternative donor splice sites.

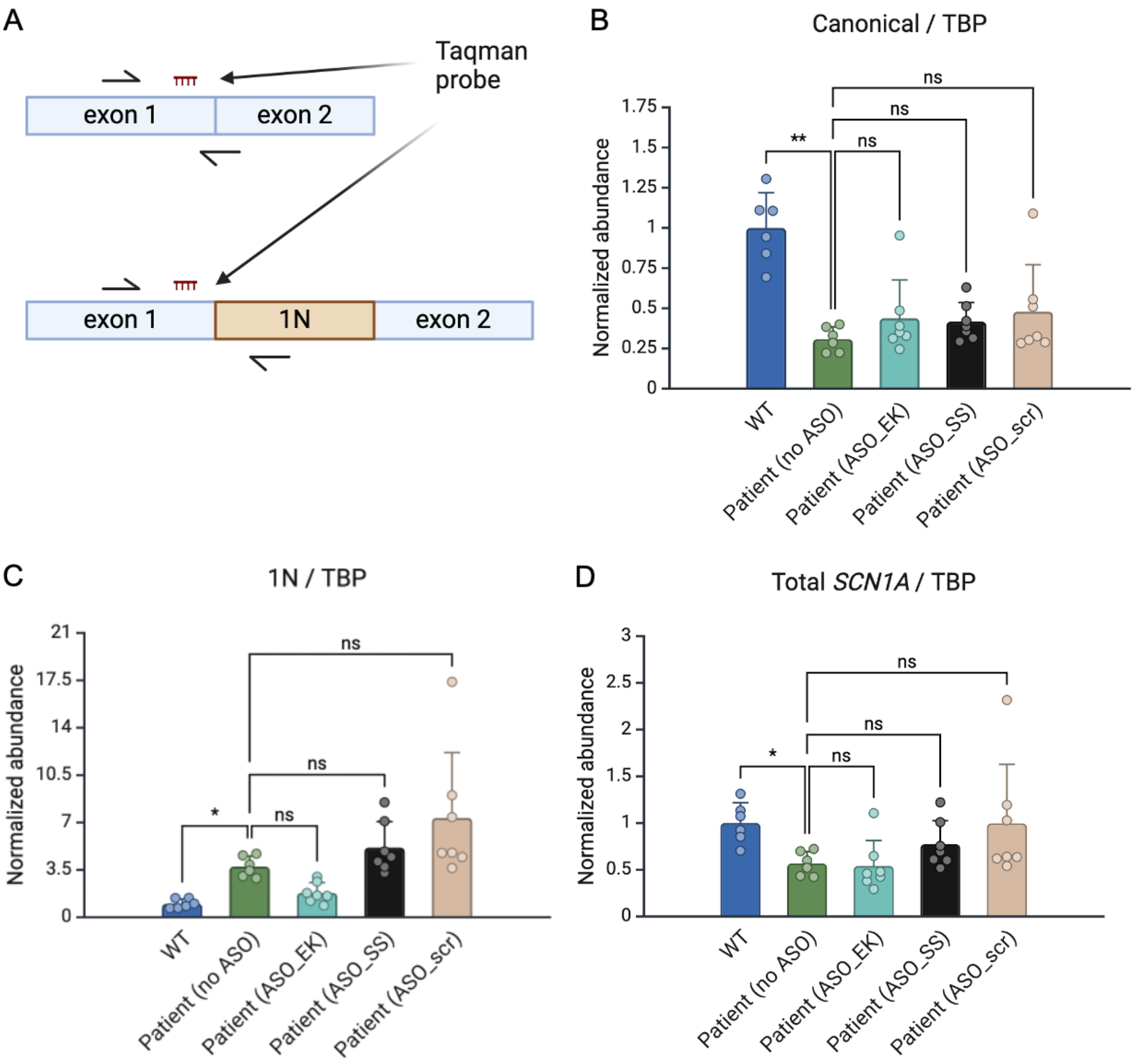

Figure S7. ddPCR results for untreated and ASO-treated iNeurons carrying the 1N-related variant.

(A) Primer and Taqman probe locations for assessing canonical or 1N-containing transcripts. Of note, the reverse primer within 1N is designed to capture all alternatively spliced isoforms of 1N. Created with BioRender.

(B) Abundance of *SCN1A* canonical transcripts. Each value (copies/uL) was first normalized to TBP (copies/uL), then normalized to the mean of samples in the WT condition (n=6-7 biological replicate wells of iNeurons per condition; Kruskal-Wallis test with Dunn's multiple comparisons test, $p = 0.006$; patient (no ASO) vs WT, $p = 0.001$; patient (no ASO) vs patient (ASO_EK), ns; patient (no ASO) vs patient (ASO_SS), ns; patient (no ASO) vs patient (ASO_scr), ns; in the patient (no ASO) condition, one sample was excluded due to a significant result on Grubb's outlier test, $p < 0.05$).

(C) Abundance of *SCN1A* 1N-containing transcripts. Each value (copies/uL) was first normalized to TBP (copies/uL), then normalized to the mean of samples in the WT condition (n=6-7 biological replicate wells of iNeurons per condition; Kruskal-Wallis test with Dunn's multiple comparisons test, $p < 0.0001$; patient (no ASO) vs WT, $p = 0.03$; patient (no ASO) vs patient (ASO_EK), ns; patient (no ASO) vs patient (ASO_SS), ns; patient (no ASO) vs patient (ASO_scr), ns; in the patient (no ASO) condition, one sample was excluded due to a significant result on Grubb's outlier test, $p < 0.05$).

(D) Abundance of total *SCN1A* 1N-containing transcripts. Each value (copies/uL) was first normalized to TBP (copies/uL), then normalized to the mean of samples in the WT condition. (n=6-7 biological replicate wells of iNeurons per condition; Kruskal-Wallis test with Dunn's multiple comparisons test, $p = 0.01$; patient (no ASO) vs WT, $p = 0.04$; patient (no ASO) vs patient (ASO_EK), ns; patient (no ASO) vs patient (ASO_SS), ns; patient (no ASO) vs patient (ASO_scr), ns; in the patient (no ASO) condition, one sample was excluded due to a significant result on Grubb's outlier test, $p < 0.05$).

Table S1. Sequences and target information for ASOs used in this study

Name	Sequence without chemical modifications	Target
SCN1A_22N_ASO1	GCTTGAAAGAGGCTGAAATC	*SCN1A* intron 22:22N junction
SCN1A_22N_ASO2	CTACATCTTACAAAGTTTTG	*SCN1A* 22N:intron 22 junction
SCN1A_22N_scr	GGCTAGGTTAAGTGACACAA	Scrambled version of SCN1A_22N_ASO2
SCN1A_1N_ASO_SS	GATGAGAGCAAAACTCCATCTGAA	*SCN1A* intron 1:1N splice junction
SCN1A_1N_ASO_EK	AAGATCGCGCCATTGCACTCCAGC	5’ end of *SCN1A* 1N, overlaps with the 1N patient variant
SCN1A_1N_ASO_scr	GTCAATCAAACAACTAGTCGGAGA	Scrambled version of SCN1A_1N_ASO_SS

Table S2. Primer and probe sequences used for qRT-PCR and ddPCR

Primer / probe name	Primer or Probe Sequence	Location	Experiment
SCN1A_test_F2	GATGACGACGAAAATGGCCC	*SCN1A* exon 1	Targeted PCR and long-read sequencing
SCN1A_test_R2	AAGGAAGCATTGGTGGGAGG	*SCN1A* exon 6	Targeted PCR and long-read sequencing
SCN1A_test_F3	GTAGCACTGTGGGACATCGG	*SCN1A* exon 17	Targeted PCR and long-read sequencing
SCN1A_test_R3	CACCAATAAACAGGTTCAAGGTGA	*SCN1A* exon 23	Targeted PCR and long-read sequencing
SCN1A_ex22_F1	GGATGGATATAATGTATGCAGC	*SCN1A* exon 22	qRT-PCR
SCN1A_22NPE:ex23_R1	GGAGTTCCACAAAGTTTTG	*SCN1A* 22N:exon 23 junction	qRT-PCR
SCN1A_ex22:ex23_F1	CCAGAAATGTGGAACTCCAG	*SCN1A* exon 22:exon 23 junction	qRT-PCR
SCN1A_ex23_R2	AGAAGGACCCAAAGATGATG	*SCN1A* exon 23	qRT-PCR
ins2_F1	GGTTCTCACTTGGTGGAAGC	Rat insulin 2 exon	qRT-PCR
SCN1A_ins2:ex22_R1	CTTTGAATGTGGCCTTGTGG	Rat insulin 2 exon:*SCN1A* exon 22 junction	qRT-PCR
22NPE_var_F1	GCCTCTTTCAAGCTCCAAGACCTAAAC	*SCN1A* intron 22 (22N)	Site-directed mutagenesis of 22N minigene
SCN1A_dd_ex1_F1	AAGATGACGACGAAAATGGC	*SCN1A* exon 1	ddPCR
SCN1A_dd_1N_R1	CCTGAGTGATGAGAGCAAAAC	*SCN1A* 1N	ddPCR
SCN1A_dd_ex1:ex2_R1	CCTTCCCTTTATTCAATACTATAAAAGTTTTCTTATTG	*SCN1A* exon 1:exon 2 junction	ddPCR
SCN1A_dd_ex1_int2 (PrimeTime probe, FAM)	CAGAGCCCCTGGAGGACCTG	*SCN1A* exon 1	ddPCR
